# An improved geomechanical model for the prediction of fracture generation and distribution in brittle reservoirs

**DOI:** 10.1371/journal.pone.0205958

**Published:** 2018-11-07

**Authors:** Jianwei Feng, Li Li, Jianli Jin, Junsheng Dai, Peng Luo

**Affiliations:** 1 School of Geosciences, China University of Petroleum (East China), Qingdao, China; 2 Laboratory for Marine Mineral Resources, Qingdao National Laboratory for Marine Science and Technology, Qingdao, China; 3 Research Institute of Petroleum Exploration & Development, Beijing, China; 4 Energy Division, Saskatchewan Research Council, Regina, Saskatchewan, Canada; Politecnico di Milano, ITALY

## Abstract

It is generally difficult to predict fractures of low-permeability reservoirs under high confining pressures by data statistical method and simplified strain energy density method. In order to establish a series of geomechanical models for the prediction of multi-scale fractures in brittle tight sandstones, firstly, through a series of rock mechanics experiments and CT scanning, we determined 0.85 *σ*_*c*_ as the key thresholds for mass release of elastic strain energy and bursting of micro-fractures. A correlation between fracture volume density and strain energy density under uniaxial stress state was developed based on the Theory of Geomechanics. Then using the combined Mohr-Coulomb criterion and Griffith’s criterion and considering the effect of filling degree in fractures, we continued to modify and deduce the mechanical models of fracture parameters under complex stress states. Finally, all the geomechanical equations were loaded into the finite element (FE) platform to quantitatively simulate the present-day 3-D distributions of fracture density, aperture, porosity, permeability and occurrence based on paleostructure restoration of the Keshen anticline. Its predictions agreed well with in-situ core observations and formation micro-imaging (FMI) interpretations. The prediction results of permeability were basically consistent with the unobstructed flow distributions before and after the reservoir reformation.

## Introduction

As an important unconventional resource and clean-burning fuel, tight sandstone gas has been widely distributed in many countries of the world, such as the U.S., Canada, Australia, Mexico, Russia, and China [1], [2], [3]. In general, tight sandstone reservoirs distinctively differ from conventional reservoirs because of their great burial depth, strong diagenesis, strong heterogeneity, abnormal overpressure, low porosity and low permeability, and developed fractures [4], [5], [6], [7]. In these tight low-permeability sandstones, the majority of reservoir spaces and seepage channels for hydrocarbon are primarily provided by widely distributed natural fractures, especially tectonic fractures, which can significantly and effectively improve the permeability of a reservoir and enhance hydrocarbon delivery to wellbores [8], [9], [10], [11], [12], [13]. Therefore, understanding and interpreting where and when tectonic fractures develop within a geological structure, along with their orientation, intensity and porosity, are important in both exploration and production of tight sandstone reservoirs. However, how to effectively predict fractures is still a worldwide challenge. Three-dimensional (3D) characterization and modeling of subsurface tectonic fractures in deep tight gas reservoirs with complex tectonism and diagenesis presents more difficulty [14], [15], [16], [17].

For exploration and production of fractured reservoirs, fracture prediction or modeling is commonly based on geometric and/or kinematic models, such as analyses of fault-related folds and fold curvature [18], [19], [20], [21], seismic techniques or logging methods [22]. These approaches, aiming to acquire the attributes of inter-well fracture networks, are useful in that they stick to the actual measured data and relate deformation to corresponding structural position [23]. Nevertheless, they are specifically tied to ideal geometric models or simplified assumptions that may not completely reflect the multi-phase deformation behaviors and mechanical properties of underground rocks [8], [14], [23].

Generally, tectonic stress field is the most important factor in controlling development and distribution of tectonic fractures in reservoirs [24], [25], [26], [21], [27]. Therefore, one efficient geomechanical modeling strategy used in recent exploration and development of brittle reservoirs is studying concentrations and changes in paleotectonic stress field so as to determine critical process involved in fracture development and combine various rupture criterions to predict favorable zones of fractures [20], [28], [29], [23], [30], [31].

Since the 1960’s, many studies have been published on the mechanisms of fracture-generating structural movement, including rock failure criterion, indicator of comprehensive rupture rate, and strain energy density. Price [32] proposed that fracture intensity was positively correlated with the elastic strain energy in rocks based on laboratory experiments. Song [33] successfully applied tensional failure criterion and shear failure criterion to calculate a rock fracturing index that predicted fracture development zones and dominant orientation. Tan and Wang [34] as well as Zhou et al. [35] put forth a similar quasi-binary method that combined failure degree value and energy degree value to quantitatively characterize fracture density. The method was based on Maximum Strain Energy Density Theory that rocks with high-strain energy possessed more fractures than rocks with low strain energy. According to the theory of strain energy density, Dai et al [36] attempted to establish a series of formulas relating stress-strain and fracture volume density and linear density under paleostress field based on comprehensive rock mechanical experiments. Olson and Laubach [14] presented a mode of natural fracture analysis that incorporates fracture mechanics and diagenetic processes to predict fracture network geometry and fracture aperture distribution and preservation in the absence of subsurface rock samples. However, almost all previous geomechanical modeling approaches for fracture prediction have been based on distributions of stress-strain, strain energy density, or rupture rate, but far less directly with further quantitative mechanical models between fracture parameters and strain energy density during different critical tectonic deformation stages. Additionally, there has been little research on the characterization of multi-scale fractures. Based on technologies of FIB-SEM tomography and X-ray μ-CT, Huang et al. [37] and Li et al. [38] conducted multi-scale quantitative characterization of 3-D pore-fracture networks in bituminous and anthracite rocks and meso-scale fracture modelling. From the perspective of evolution, Ghamgosar and Erarslan [39] used CT scan technique to investigate and evaluate the micro-fracturing samples in fracture process zones under cyclic and static loadings. The following conclusions are drawn from this study: intergranular cracks are formed due to particle breakage under cyclic loading compared with smooth and bright cracks along cleavage planes under static loading, and the macro-scale main crack causing failure is seen in cement without any dust or debris material under monotonic loading.

In this study, we first conducted rock mechanical tests and advanced X-ray CT scanning to accurately determine the key threshold values for multi-scale fracture generation and development in brittle tight sandstones. A quantitative relationship between strain energy density and fracture volume density was established. Applying the classic geological mechanics theories, i.e., the Maximum Tension Theory and Maximum Strain Energy Density Theory, we continued to deduce the associated mechanical models of fracture parameters (fracture linear density, aperture, porosity and permeability) under the complex stress states. Then, on the basis of tectonic evolutionary restoration of Keshen anticline in the Kuqa Depression, we used finite element method (FEM) to better simulate 3-D paleostress field and current stress field during the entire deformation process of faults and folds. Combining with composite fracture criterion and considering the influences of the current stress and filling degree on the aperture of a fracture, we incorporated these mechanical models into a numerical simulator ANSYS to predict the distributions of tectonic fractures in Keshen tight gas field, which were effectively verified by the measured fracture density from drilled cores and imaging logs.

## Geologic setting

**Fig 1:**
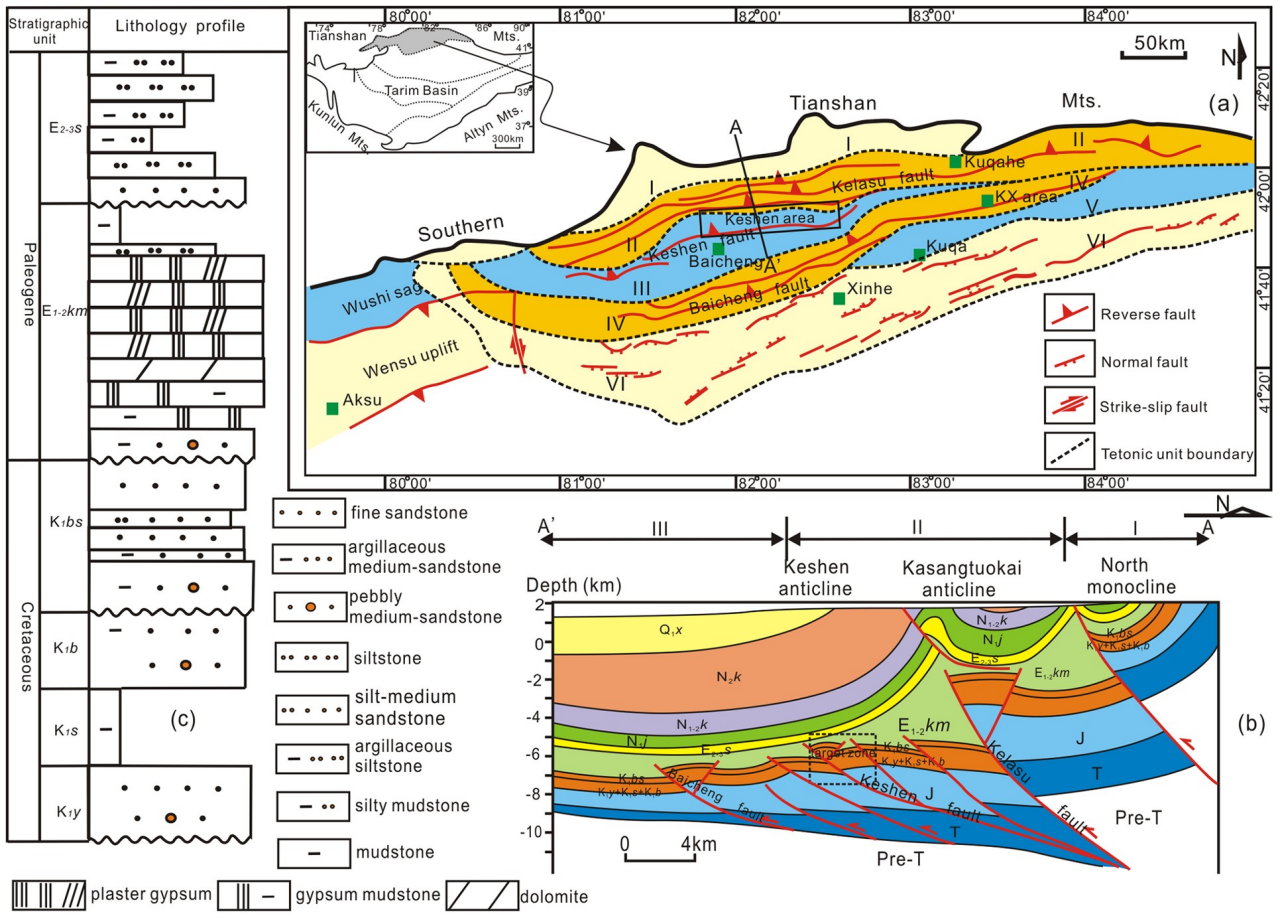
Maps show the elementary structural features of the Kuqa Depression. (a) Location of the study area (from Zhang et al., [42]), I-Northern monocline tectonic zone; II-Kelasu tectonic zone; III-Baicheng sag; IV-Qiulitage tectonic zone; V-Yangxia sag; VI-Northern Tarim Uplift; (b) Tectonic cross-section of the location shown in (a). Pre-T: Silurian to Triassic; J: Jurassic; K_1_ *y*-K_1_ *s*-K_1_ *b*: Yageliemu Formation-Shushanhe Formation-Baxigai Formation; E_1-2km_: Kumugeliemu Formation; E_2-3s_: Suweiyizu Formation; N_1j_: Jidike Formation; N_1k_: Kangcun Formation; N_2k–Q1x_: Kuqa Formation to Quaternary; (c) Schematic stratigraphy of the Kuqa Depression.

The Kuqa Depression is located at the northern margin of the Tarim Basin between the South Tianshan Orogenic Belt and the Northern Tarim Uplift (Fig 1). Structures that develop widely in the Kuqa Depression during the Cenozoic Period are dominated by thrust faults and related folds. Laterally, the Kuqa Depression can be divided into three structural belts and two sags, which are, from north to south, the northern monocline belt, the Kelasu structural belt, the Baicheng sag, the Kuqa depression, and the Qiulitage structural belt (Fig 1a) [40], [29]. The Kuqa Depression is recognized as one of the major Cenozoic depocenters along the margin of the Tarim Basin because it has experienced a complex evolution since the Late Cretaceous with the northward movement of the Indian sub-continent and the southward thrusting of the South Tianshan [41], [42], [43]. The Keshen gas field is situated in the footwall of Kelasu tectonic belt, south of Kela fault, and displays a narrow anticline with a structure amplitude of less than 500 m. The top of the Keshen gas field is cross-cut by several faults striking E-W to become complicated, acquiring a dip in the southern wing ranging from 16° to 20° and in the northern wing ranging from 19° to 23° (Fig 1b).

Fracture intervals in the Keshen gas field consist of Cretaceous delta front rocks (the Bashijiqike Formation, K_1_ *bs*), which are dominated by fine sandstone, siltstone, and mudstone with limited sandy conglomerate. These beds reach a total thickness of up to 290m within the study area and are divided into three members according to lithological cycles and interbeds (Fig 1c), [29], [7]. The porosity of the Bashijiqike reservoir, as determined by core tests, ranges from 2–7%. Permeability of the reservoir lies in the range of (0.05–0.50) ×10^−3^ μm^2^, while fracture permeability can reach (1.00–10.00) ×10^−3^ μm^2^. In summary, the above evidence indicates that the Keshen gas field belongs to a typical ultra-deep low-porosity and low-permeability tight sandstone reservoir.

**Fig 2:**
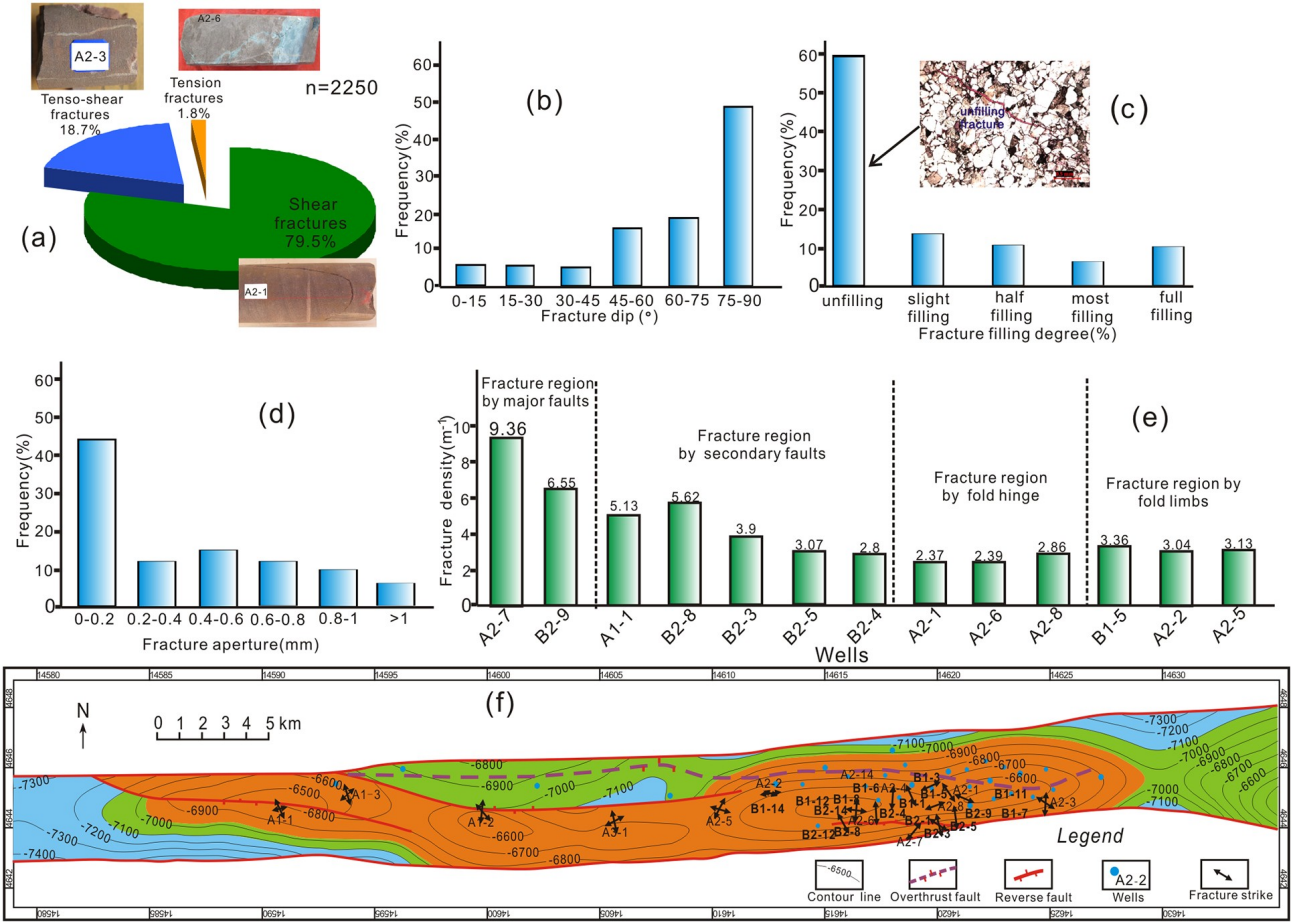
Characteristics of tectonic fractures observed in drill cores. (a) Statistical analysis of the types of fractures; (b) Statistics of fracture dip observed in all wells; (c) Statistics of fracture filling degree observed in all wells; (d) Statistics of fracture aperture observed in all wells. (e) Statistics of fracture linear density observed in different zones; (f) Structural map of the top Lower Cretaceous Bashijiqike Formation in the Keshen gas field, with predominant strikes of fractures obtained from orientated cores.

Based on quantitative observation and statistical description in drilling cores and formation micro-imaging (FMI), simultaneously using field outcrops for reference, the fractures most frequently encountered in the reservoir of the area are planar discontinuities that are sub-perpendicular to the bedding and can be divided into three basic types, namely tension fracture, tenso-shear fracture, and shear fracture, respectively accounting for 79.5%, 18.7%, 1.8% of the total fracture volume (Fig 2a). The shear fracture has a straight rupture plane, a longer extended distance than the other two types, and in most cases can cut through rock grains. The tensional fracture has a dendritic structure, a relatively shorter distance, and frequently bypasses rock grains, wherever they are observable by drill core and FMI. Observations from cores and analysis from FMI show that dip of fractures mainly ranges from 75°– 90° (i.e. vertical fractures), followed by 45°–75° (i.e. high-angle fractures) and 15°–45° (i.e. low-angle fractures) (Fig 2b). Due to the effect of stress unloading after exposure to ground, the physical parameters of fracture network will change slightly relative to underground conditions, such as fracture aperture and porosity. With fine integration of core observation and well-logging data, the detailed parameters of tectonic fractures can be obtained. It is shown that most fracture in K_1_ *bs* are unfilled and slight-filled, which accounts for more than half of the total fracture number (approximately 72%) (Fig 2c). In contrast, only 17% of total fractures are most-filled and full-filled, with calcite and dolomite as the main fillings, followed by small amounts of mud and carbon. The statistical result shows that the fracture aperture in this study area has a bimodal distribution with the main peak range of 0–0.2 mm and 0.4–0.6 mm, and the former dominated in addition (Fig 2d).

The fracture density generally contains linear density (1/m) and volume density (m^2^/m^3^), and the former is an important parameter to illuminate the development and distribution characteristics of fractures [21]. Observations made in the drilling cores of the tight sandstones in the limbs and top of the anticline show obvious relationship between fractures and structural positions. Vertical fractures and echelon fractures mainly distribute in the fold hinge area, with linear density ranging from 1.2/m to 1.5/m and length and vertical extent often greater than 10 m (Fig 2e). Structures in two limbs are mainly composed of high-angle conjugated fractures with linear density reaching up to 2.33/m, that is characteristic of systematic joints as defined by Engelder [44]. On the whole, the fracture density has higher value in limbs than that of hinge area and has higher value in southern limb than that of northern limb. However, near the fault zone, there has the highest fracture density value (9.36/m), which can be called the fracture damage zone (Fig 2e and 2f).

Based on the above statistics, the fractures present in the Bashijiqike Formation can be subdivided based on orientation into four distinct, mutually abutting fracture sets oriented NW-SE (set I), NNE-SSW (Set II), NE-SW (set III) and nearly EW (set IV), among which the former two sets are dominant (Fig 2f).

## Experiments

### Core samples

Lithologically, K_1_ *bs* member in the Keshen gas field were dominated by lithic feldspar sandstone, followed by feldspar lithic sandstone and little feldspathic sandstone. The sandstones are medium grained (0.5–0.25 mm) and fine grained (0.25–0.05 mm), medium cemented, medium to well sorted, containing much cement between the particles. The mineralogical composition of sandstone is 50% quartz, 12% feldspar, 5% clay, and 33% debris, respectively. This composition indicates that the K_1_ *bs* belonged to medium hard to hard brittle sandstone with strong elastic-brittle characteristics. Forty-eight core plugs were drilled from K_1_ *bs* along the direction of the bedding plane. All plugs were processed according to ISRM-suggested methods with a core diameter of 25 mm and length of 50 mm, ends ground flat to 0.01 mm with vertical deviation of less than 0.25°.

### Rock mechanical measurements

As listed in Tables 1 and 2, the plugs were subjected to a series of rock mechanical experiments that included uniaxial compression tests, triaxial compression tests, direct shear tests, and splitting tests. A Uniaxial/Triaxial Mechanics Instrument (TAWA-2000, USA) was used to simulate the failure process of plugs. This is a hybrid loading instrument with a maximum axial pressure of 2,000 kN and a maximum confining pressure of 140 MPa used for uniaxial compression and triaxial compression tests, with a loading rate of 0.01 mm/s. Over the course of loading, the confining pressure was manually set to seven stages at an interval of 5 MPa (0, 5, 10, 15, 20, 25 and 30 MPa) as the axial pressure increased up to peak stress. At all times, axial loads exceeded confining pressure by no more than one-tenth of the rock uniaxial compression strength (UCS). Before the CT scanning tests, uniaxial compression tests of several spare samples were conducted to make estimation of uniaxial compression strength or peak strength (σ_c_). In this way, the complete stress-strain curves and mechanical parameters of sandstone were acquired, including the shear strength (*τ*_*s*_), cohesive strength (*C*_0_), and friction angle (*φ*) (Tables 1 and 2) (S1 Appendix).

**Table 1:** Experimental datasets from uniaxial compression tests.

Well	Sample No.	Rock type	Depth (m)	ρ (g/cm^3^)	*σ*_*c*_ (MPa)	E (GPa)	G (GPa)	*μ*	Failure mode
**A2-2**	C1	Siltstone	6765.2	2.62	30.28	4.05	1.94	0.045	Tenso-shear
**A2-2**	C2	Mid-fine sandstone	6765.4	2.53	50.51	6.36	2.98	0.069	Tenso-shear
**A2-2**	C3	Mid-fine sandstone	6765.42	2.55	34.0	3.45	1.95	0.058	Tension
**A2-1**	C4	Silty sandstone	6707.01	2.69	43.55	5.03	2.32	0.082	Tension
**A2-1**	C5	Calcareous siltstone	6707.32	2.63	67.18	12.78	7.77	0.082	Tension
**A2-1**	C6	Calcareous siltstone	6707.43	2.60	73.68	12.08	7.00	0.148	Tension

**Table 2:** Experimental datasets from triaxial compression tests.

Well	Sample no.	Depth (m)	Confining pressure (MPa)	*σ*_*c*_ (MPa)	*E* (GPa)	*G* (GPa)	*μ*	Cohesive strength *C*(MPa)	Friction angle *ϕ* (°)
**A1-1**	T1	6941.8	5	39.85	3.98	2.78	0.103	7.40	39.64
	T2	6942.9	10	47.44	4.15	3.39	0.166		
	T3	6942.1	15	54.25	4.26	3.73	0.194		
**A2-1**	T4	6711.2	20	44.94	4.12	8.75	0.134	8.16	38.40
	T5	6711.3	25	53.66	5.54	9.71	0.200		
	T6	6711.5	30	60.68	7.08	6.18	0.213		
**A2-2**	T7	6767.2	20	55.63	3.08	7.30	0.158	9.98	35.73
	T8	6767.4	25	74.61	4.33	7.54	0.216		
	T9	6767.5	30	79.86	6.81	6.80	0.267		

### CT Scanning experiments

**Fig 3:**
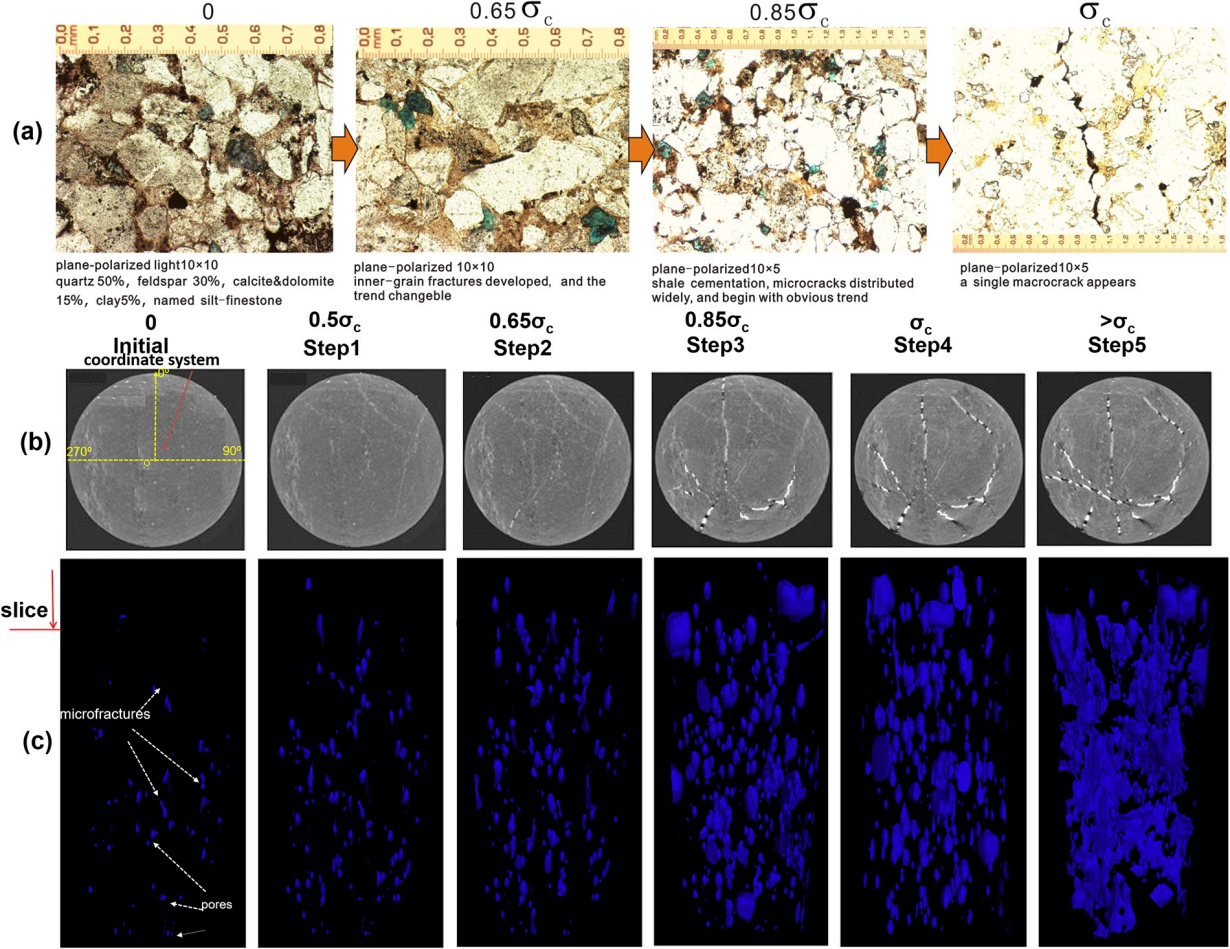
Evolution diagram of micro-cracks in tight sandstone sample (C5) under uniaxial compressional tests based on CT scanning and microscopic observation. (a) Thin slices of micro- and macro-cracks parallel to the rock sample cross-section, viewed under plane polarized light. (b) Sliced CT images of middle layer during different compression stages and coordinate system for fracture parameter measurement. (c) 3D-crack images are reconstructed from fine scans of inner cracks conducted via CT scanner with a precision of less than 1 μm at different loading stages. Note: In order to avoid sudden collapse of the rock, a low confining pressure 0.1 MPa was imposed on the external tube of Mechanics Instrument in the whole process of test.

The CT (Computer Tomography) scanning experiments were conducted at a Micro XCT-400 CT scanner at the Research Center of Oil & Gas Flow in Reservoir, China University of Petroleum. The mechanical deformation process of six core samples under classical uniaxial tests was monitored real time by the CT scanner (Fig 3). As shown in Fig 3a, the core compression device was made of beryllium that has low attenuation under X-ray. During the multi-stage loading test, the sample was loaded to various percentages of the peak stress (i.e., peak strength σ_c_) acquired from the preliminary uniaxial tests, and the crucial load increment between two cycles was determined by each rapid generation of micro-fractures during uniaxial compression processes. Seven scans were performed in the loading process, i.e., at the initial loading, 25%, 50%, 65%, 85%, 100%, and 110% of stress peak, and post-peak, respectively, with a CT slice thickness of 2 mm, 25 slices per scan (S2 Appendix). For a grey-level CT image, the bright color denotes the low-density part such as fractured zone or pores, and the dark color denotes the high-density rock matrix, as shown in Fig 3b. The scanned 2-D images were reconstructed into 3-D geometry and then imported to Avizo (ThermoFisher Scientific, USA) for further image analysis (Fig 3c). In this study, it is important to distinguish induced different-sized fractures and natural vugs from CT images. As irregular morphology (e.g., nearly round, calabash-shaped or curviplanar) and various sizes after image processing, the micro-scale pore-throats were calculated to be 5–90 μm in diameter and primarily distributed in isolated points, and locally in strips (Fig 3c). In contrast, the original micro-fractures developed in intact rock samples were measured to be 5–15 μm wide and 300 μm long, mainly took on three kinds of shapes: short intragranular fracture, straight rectangular fracture, and crooked grain-edged fracture with sharp boundaries between grains. Then, the fractures and vugs in processed CT images at initial loading stage could be easily classified with visual observation, and the original vug volume was necessarily subtracted from subsequent total damage or fracture volume during each deformation stage.

Finally, the strain energy density and different-scale fracture density of the low-permeability tight sandstone samples corresponding to each crucial fracture growth stage were calculated, described in detail, and translated into a series of stress-strain curves for the purpose of identifying relationships between stress and fracture parameters (Table 3).

**Table 3:** Random sampling results of stress-strain state and fracture parameters of tight sandstones in uniaxial experiments. Note: For scanning efficiency, the volume density and aperture of micro-fractures before appearance of macro-fractures was defined as zero here.

Sample No.	Stress at sampling *σ*_*Q*_ (MPa)	Bearing ratio $\frac{\sigma_{Q}}{\sigma_{c}}$	Axial deformation ratio $\frac{\varepsilon_{h}}{\varepsilon_{\text{hc}}}$	Radial deformation ratio $\frac{\varepsilon_{r}}{\varepsilon_{\text{rc}}}$	Volume Density (m^2^/m^3^)	Average aperture (mm)	Stain energy density (J/m^3^)
**C1**	25.43	0.84	1.1	0.95	22.30	0.0240	1.358E+05
**C2**	43.55	1.02	1.02	1.38	43.00	0.0280	1.619E+05
**C3**	34.0	0.8	1.1	0.95	22.50	0.0240	1.356E+05
**C4**	50.52	1.18	0.88	0.67	40.35	0.0020	1.618E+05
**C5**	47.36	0.88	0.91	1.10	13.40	0.0180	1.242E+05
**C6**	23.18	0.54	0.78	0.35	0.00	0.0000	6.592E+04

## Results analysis

### Evolution of fractures under uniaxial compression

**Fig 4:**
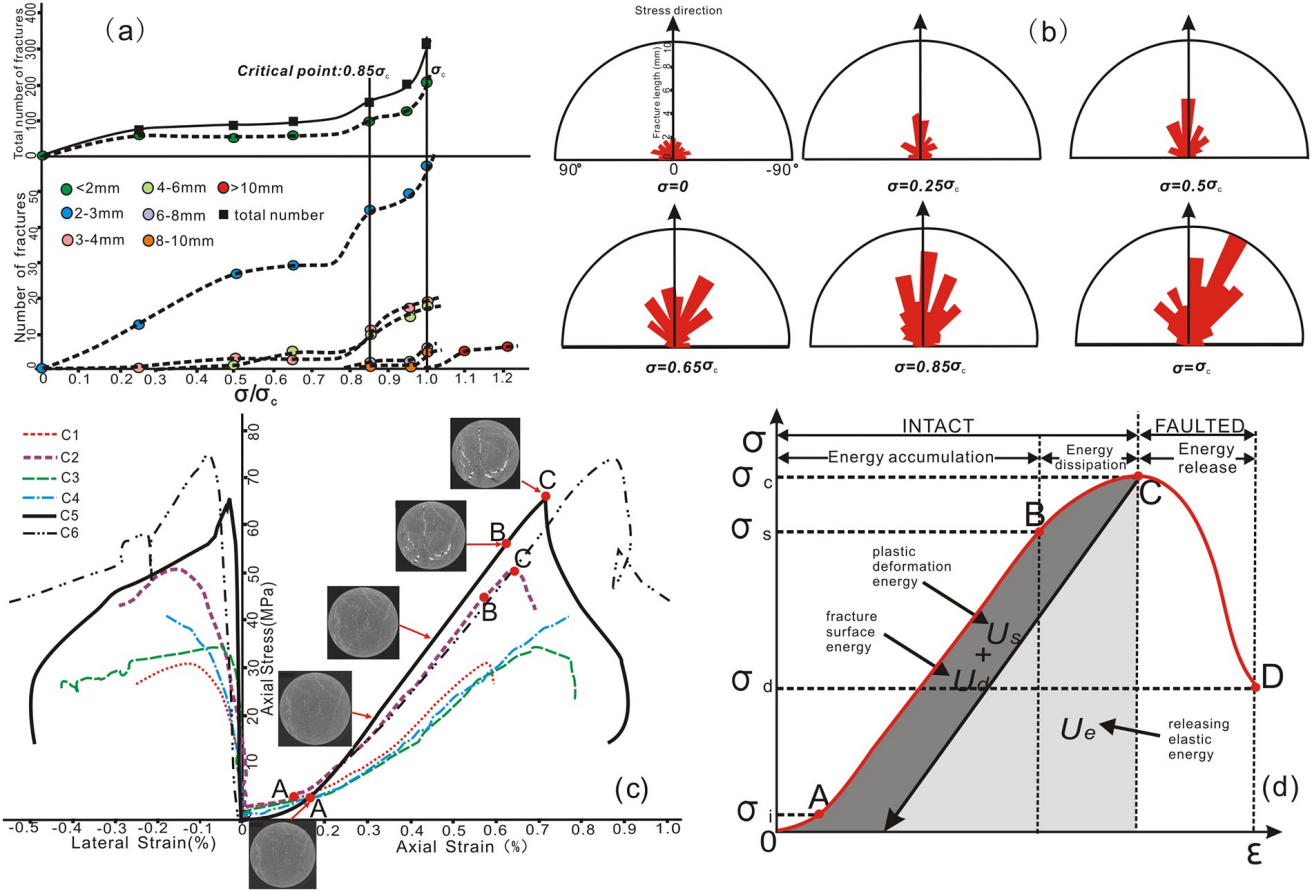
Evolution modes for fractures of samples C3 and C5 under loading process. (a) Number evolution of different-scale fractures during critical deformation stages. (b) Rose diagrams showing the variation of average fracture length with direction. (c) Uniaxial stress-strain curves of various brittle sandstone samples. (d) A diagram showing the stages of rock deformation in uniaxial compression tests.

According to the statistical results in Fig 4a, at the beginning the pre-existing micro-fractures in rocks began to close with compression, allowing rocks to become dense, fracture aperture to reduce significantly. In this stage, most of the original micro-fracture length was less than 0.3 cm. When the applied stress was 25% and 50% of the peak value, a few small new micro-fractures with random orientations began to initiate, among which the fractures with length ranging from 0.2 cm to 0.3 cm had relatively higher growth rate than other fractures of different scales. When the applied stress was increased to 65% of the peak value, growth rate difference gradually appeared among all different-scale fractures, such as the number of small-scale micro-fractures (length<0.3 cm) still steadily increased, whereas that of relatively middle-scale fractures (length ranging from 0.4–0.6 cm) rapidly increased at this stress stage, and that of fractures with length ranging from 0.3 to 0.4 cm decreased instead. Undoubtedly, new generating fractures in this stage tended to grow preferentially in the stress direction. When the applied stress was increased to 85% of the peak value even after this point, there was a rapid increase in the number of all scales of fractures (except the length<1 cm), which were of strong orientation and approximately equal in length. At the same time, after this critical stress value, large-scale fractures (length exceeding 1cm) began to appear, and their number gradually increased. This indicated that fractures tended to develop along anisotropy when the angle between pre-existing micro-fractures and stress direction was low (<0°) (Fig 4b). However, during these stages, apertures of most of the fractures were still less than 100 μm, and they belonged to micro-scale fractures. When the applied stress was increased to the peak value or even after this point, it was interesting that only the number of large-scale fractures (length even reaching 5 cm and aperture even exceeding 100 μm) suddenly increased, in contrast, the other levels of fractures were almost invariable in number. It could be seen clearly for Figs 3 and 4 that these seemingly randomly distributed micro-fractures formed in previous stages began to coalesce with one another along certain predominant direction until the formation of macro-fractures, which further propagated along the stress direction approximatively (Fig 4b).

From the above evidence, it could be concluded that a large number of new fractures appeared at the compression stress levels of approximately 0.85 σ_c_ (i.e., point B in Fig 4c and 4d), which could be defined as the threshold values for micro-fracture inception and development during stress loading. Zhao [45] conducted uniaxial compression experiments on Mechanics Instrument with sandstone samples under various stress conditions and find that micro-fractures increases as stress boosted, especially when stress reaches or exceeds 0.85 σ_c_ or 0.88 σ_c_. At this stress level, the number of fractures exploded instantaneously and the number of middle-sized fractures (0.6 cm>length>0.2 cm) increased faster than that of small-scale and large-scale fractures. At the same time, the number of fractures with small angles with respect to principal stress (≤30°) increased faster than that of fractures with larger angles (>30°) (Fig 4b). While the axial stress reached peak strength at Point C, the overall macro-rupture occurred along with a significant decrease in stress and a significant increase in strain (Figs 4c and 4d). When the stress exceeded the peak strength of rock sample, it began to fall until the residual strength remained, thus the structural fault was created, accompanied by a large amount of frictional energy.

### Geomechanical modeling of fracture density under uniaxial stress state

In Earth’s crust, when a rock is subjected to a 3-D stress condition, the strain energy density at a given point within the rock mass can be expressed as follows [46], [21]:

$$\varpi \;=\;\frac{1}{2E}[{\sigma_{1}}^{2}+{\sigma_{2}}^{2}+{\sigma_{3}}^{2}-2\mu (\sigma_{1}\sigma_{2}+\sigma_{2}\sigma_{3}+\sigma_{1}\sigma_{3})]$$ (1)

where *σ*_*1*_, *σ*_*2*_ and *σ*_*3*_ were the maximum principal stress, intermediate principal stress and minimum principal stress, respectively (MPa); *E* was the elastic modulus and μ was the Poisson’s ratio.

According to brittle fracture mechanics theory and maximum tensile stress theory, brittle rock will break when elastic strain energy accumulated in the brittle material equals to the energy demanded for generating fractures per unit volume of the element [47], [48]. Generally, brittle macro-fractures occur with strain energy releasing, especially when the surrounding three-dimensional stress state reaches the rock’s strength [49]. At this time, part of the strain energy will be released as the surface energy of the new fractures while the rest will be released in form of elastic waves. Nonetheless, compared with the fracture surface energy, elastic wave energy was so weak that it can be neglected. We assume that all of the fractures in this study were caused by the releasing energy and therefore (Fig 4d), the law of conservation of energy must be satisfied:

$$D_{vf}\;=\;\frac{S_{f}}{V}\;=\;\frac{\varpi_{f}}{J}\;=\;\frac{\varpi -\varpi_{e}}{J}\;=\;\frac{1}{J}\varpi -\frac{\varpi_{e}}{J}\;=\;a\varpi +b$$ (2)

where *D*_*vf*_ was the fracture volume density per unit volume (m^2^/m^3^); *ϖ_f_* was the strain energy density for new fractures (J/m^3^), also was regarded as the residual strain energy density by current strain energy density per unit volume minus the elastic strain energy density for new fractures (*ϖ_e_*) (J/m^3^); *V* was the unit cell volume (m^3^); S was the surface area of the new fractures (m^2^); *J* was the energy per unit area required for fractures, i.e., fracture surface energy (here, this energy was different from and had a far lower value than the theoretical value of molecular dissociation); coefficients $a=\frac{1}{J}$, and $b=-\frac{\varpi_{e}}{J}$ were relative coefficients, which could be derived by two methods: (1) Based on the values of *J*, *ϖ_e_* was calculated by physical concept and then *a* and *b* were obtained through conversion values. (2) Through experimental data, curve fitting and a statistical approach were employed to obtain the coefficients. In practice, the former has clearer physical meaning.

In most cases, there exist three types of stress environments in Earth’s crust: Type Ia, Type II, and Type III and different mechanical behaviors appear under different stress environments [50]. As stated above, with a uniaxial compressive strength level of 0.85 σ_c_ as the peak period for the initiation of fracture swarm, the corresponding strain density energy was close to *ϖ_e_* in theory. In this case, we assumed that *ϖ_e_* is strain energy density at σ = 0.85σ_c_. This value would be entered in a formula to calculate *J* and then the values of *a* and *b* could be confirmed. Based on the preceding mechanical experiments on tight sandstone in the Keshen area, the average uniaxial compressive strength of predominant mid-fine sandstone samples was identified, and we calculated ϖ_e_ as 1.133 × 10^3^ J/m^3^. Here, Eq (2) could be transformed into $J\;=\;\frac{\varpi -\varpi_{e}}{D_{vf}}$. Calculated values of *J* were presented in Table 4.

**Table 4:** Strain energy parameters of two specimens in the Keshen area. Note: *σ*_*i*_ is initial stress, *ε*_*i*_ is initial strain, *σ*_*Q*_ is stress at sampling, and all stresses are in MPa.

Sample No.	*σ*_*Q*_ (MPa)	*ε*_*Q*_ × 10^−4^	*ϖ* (J/m^3^)	*σ*_*i*_ (MPa)	*ε*_*i*_ × 10^−4^	*ϖ_e_* (J/m^3^)	*D*_*vf*_ (m^2^/m^3^)	*J* (J/m^2^)
C2	43.55	74.37	1.619E+05	36.28	62.46	1.133E+05	44.7	1087.25
C4	50.52	64.07	1.618E+05	36.28	62.46	1.133E+05	44.6	1087.44

Averaging *J* in Table 4 as $\overset{-}{J}\;=\;1055.27\frac{J}{m^{2}}$ and substituting $\varpi_{e}\;=\;1.133\times \frac{10^{3}J}{m^{3}}$ into Eq (2), a quantitative relationship between strain density and fracture volume density was developed:

$$D_{vf}\;=\;9.2\times 10^{-4}\varpi -104.2$$ (3)

**Fig 5:**
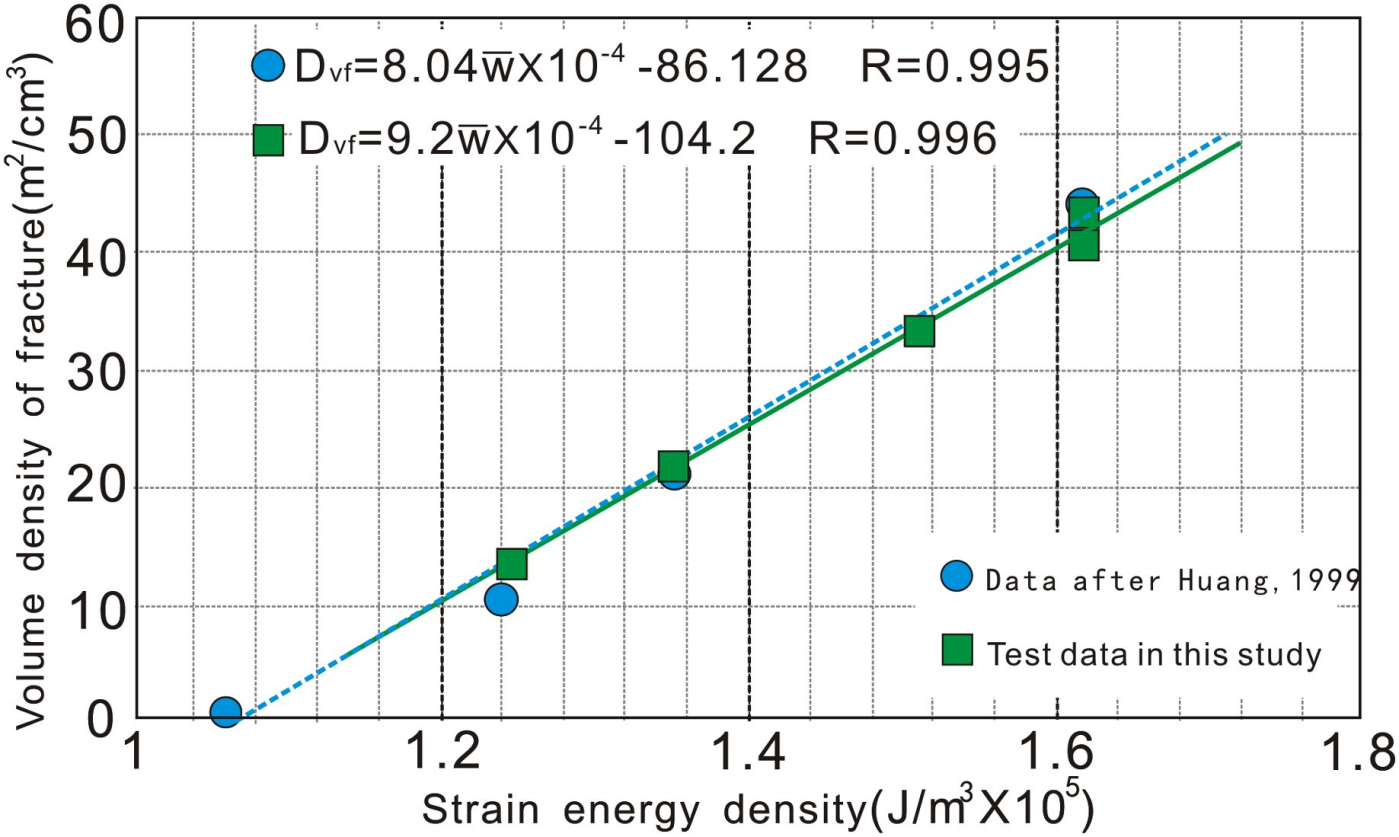
Linearized relational plot between strain energy density and fracture volume density at the key stress point of 0.85σ_c_, based on stress–strain curves. Note good agreement between these results and those of Huang [51].

To test and verify the reliability of *ϖ_e_*, a curve-fitting method was employed to calculate *a* and *b*. A comparison plot of the curve-fitting results versus data obtained by the mechanical experiments (Table 3 **)** was shown in Fig 5, from which the following linear relationship was obtained:

$$D_{vf}\;=\;8.04\times 10^{-4}\varpi -86.128$$ (4)

where the correlation coefficient reached 0.995, and the relative errors for coefficients *a* and *b* by two different methods were 12.6% and 17.3%, respectively, which are both within the permitted error range. It should be noted that regardless of whether the rock mass is intact or fractured, the fitting curve exhibited a positive linear relationship between parameters. Small deviations were explained by anisotropic mechanical properties, i.e., a combination of pre-existing weakness planes and unruptured rocks. Therefore, it was reliable that the necessarily overcoming elastic strain energy density for new fractures (*ϖ_e_*) could be replaced by the corresponding strain energy density at 0.85σ_c_ both in theory and practice.

### Geomechanical modeling of fracture parameters under paleostress field

#### Fracture geometric parameters

**Fig 6:**
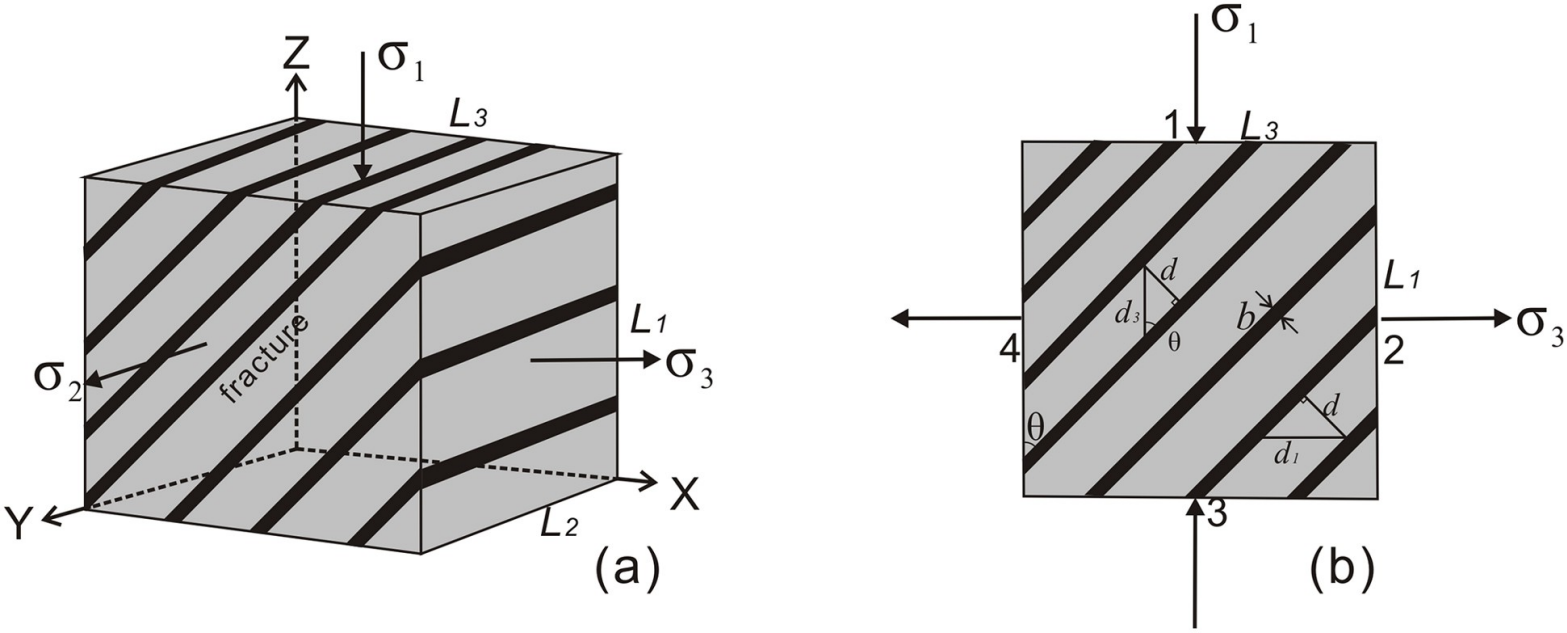
In-situ stress coordinate system representing fracture distribution and differential stress plane based on an Element volume. (a) Diagram of representative elementary volume (REV); (b) Transection in REV perpendicular to σ_2_, namely (σ_1_-σ_3_) plane. *L*_1_, *L*_2_ and *L*_3_ are the lengths (m) in the directions of *σ*_1_, *σ*_2_, and *σ*_3_, *θ* is angle between fracture plane and *σ*_1_, *b* is the fracture aperture.

As Huang [51] and Song [33] demonstrated previously, the relationship between fracture volume density and strain energy density under triaxial compression experiments is no longer presented as a regular linear relationship. In other words, the energy accumulated in the brittle rocks must not only overcome the molecular internal cohesive forces, but also overcome the energy expenditure that naturally occurs to resist the confining pressure [51], [33]. Based on the above geomechanical model under uniaxial compression, the relationships between fracture volume density and strain energy density under triaxial stress state, tensile stress state were derived as follows:

1. Commonly, tectonic fractures are divided into tensile and shear fractures based on the formation mechanism, and they can be discriminated with the Griffith Criterion and Coulomb-Navier Criterion (also called the Mohr-Coulomb Criterion), respectively [52], [33], [53], [13]. For the fractures under confining pressure conditions, if there existed only compressive stresses (*σ*_3_ ≥ 0), the Mohr-Coulomb criterion would be selected, which was
  $$\frac{\sigma_{1}-\sigma_{3}}{2}\geq C_{0}cos\;\phi +\frac{\sigma_{1}+\sigma_{3}}{2}sin\;\phi$$ (5)
  The Mohr-Coulomb Criterion suggested that a shear fracture only formed if the rock cohesion (*C*_0_) was exceeded, which was depended on the magnitude of normal stress along a fracture plane. The relationships between fracture density, aperture and strain energy density, stress-strain were written as follows
  {wf=w-we=12(σ1ε1+σ2ε2+σ3ε3)-12Eσt2Dvf=wfJJ=J0+ΔJ=J0+σ3bDlf=2DvfL1L3sinθcosθ-L1sinθ-L3cosθL12sin2θ+L32cos2θDlfb=|ε3|-|ε0|ε0=σtE (6)
  Where *ϕ* was the internal friction angle (°); *θ* is the angle between normal to the newly formed fracture plane and the maximum principal stress (°) (Fig 6a); *σ*_1_ was the maximum principal stress (MPa); *σ*_2_ was the intermediate principal stress (MPa); *σ*_3_ was the minimum principal stress (MPa); *σ*_*p*_ was the rupture stress under action of *σ*_3_, different from the maximum principal stress; *σ*^*t*^ was the tensile strength (MPa); *J*_0_ was fracture surface energy with no confining pressure or under uniaxial compressive stress (J/m^2^), Δ*J* was the additional surface energy caused by confining pressure *σ*_3_ (J/m^2^); *E* was the elasticity modulus with no confining pressure (GPa); *b* was the fracture aperture (i.e. paleo-aperture) (m), referring to Fig 6b; *D*_*If*_ was the fracture linear density (1/m); *ε*_3_ was the tensile strain under current state of stress, dimensionless parameter; *ε*_0_ was the maximum tensile strain, dimensionless parameter, corresponding to tensile strain when fracture beginning to form; *E*_0_ was the proportionality coefficient related to lithology; and *L*_1_, *L*_2_, *L*_3_ were side length of the selecting representing element volume (Fig 6a).
2. When there existed tensile stress, for brittle tight sandstone material the Griffith Criterion was used. When *σ*_3_ ≤ 0 and (*σ*_1_ + 3*σ*_3_) ≥ 0, the applied failure criterion was given as
  $$(\sigma_{1}-\sigma_{2}{)}^{2}+(\sigma_{2}-\sigma_{3}{)}^{2}+(\sigma_{3}-\sigma_{1}{)}^{2}\geq 24\sigma_{T}(\sigma_{1}+\sigma_{2}+\sigma_{3}),cos\;2\theta \;=\;\frac{\sigma_{1}-\sigma_{3}}{2(\sigma_{1}+\sigma_{3})}$$ (7)
  When (*σ*_1_ + 3*σ*_3_) < 0, the failure criterion was simplified to *σ*_3_ = −*σ*_*T*_, *θ* = 0.

If the failure criterion was reached, the relationships between fracture density, aperture and strain energy density, stress-strain were expressed as

{wf=w-we=12(σ1ε1+σ2ε2+σ3ε3)-12Eσt2Dvf=wfJJ=J0+ΔJ=J0+σ3bDlf=2DvfL1L3sinθcosθ-L1sinθ-L3cosθL12sin2θ+L32cos2θDlfb=|ε3|-|ε0|ε0=σtE (8)

According to the above description, when (*σ*_1_ + 3*σ*_3_) > 0, by Griffith criterion, there should be: $cos\;2\theta \;=\;\frac{\sigma_{1}-\sigma_{3}}{2(\sigma_{1}+\sigma_{3})}$ and $\theta \;=\;\frac{arccos\;\left[\frac{\sigma_{1}-\sigma_{3}}{2(\sigma_{1}+\sigma_{3})}\right]}{2}$, and when (*σ*_1_ + 3*σ*_3_) ≤ 0, *θ* = 0, we have *D*_*If*_ = *D*_*vf*_.

**Fig 7:**
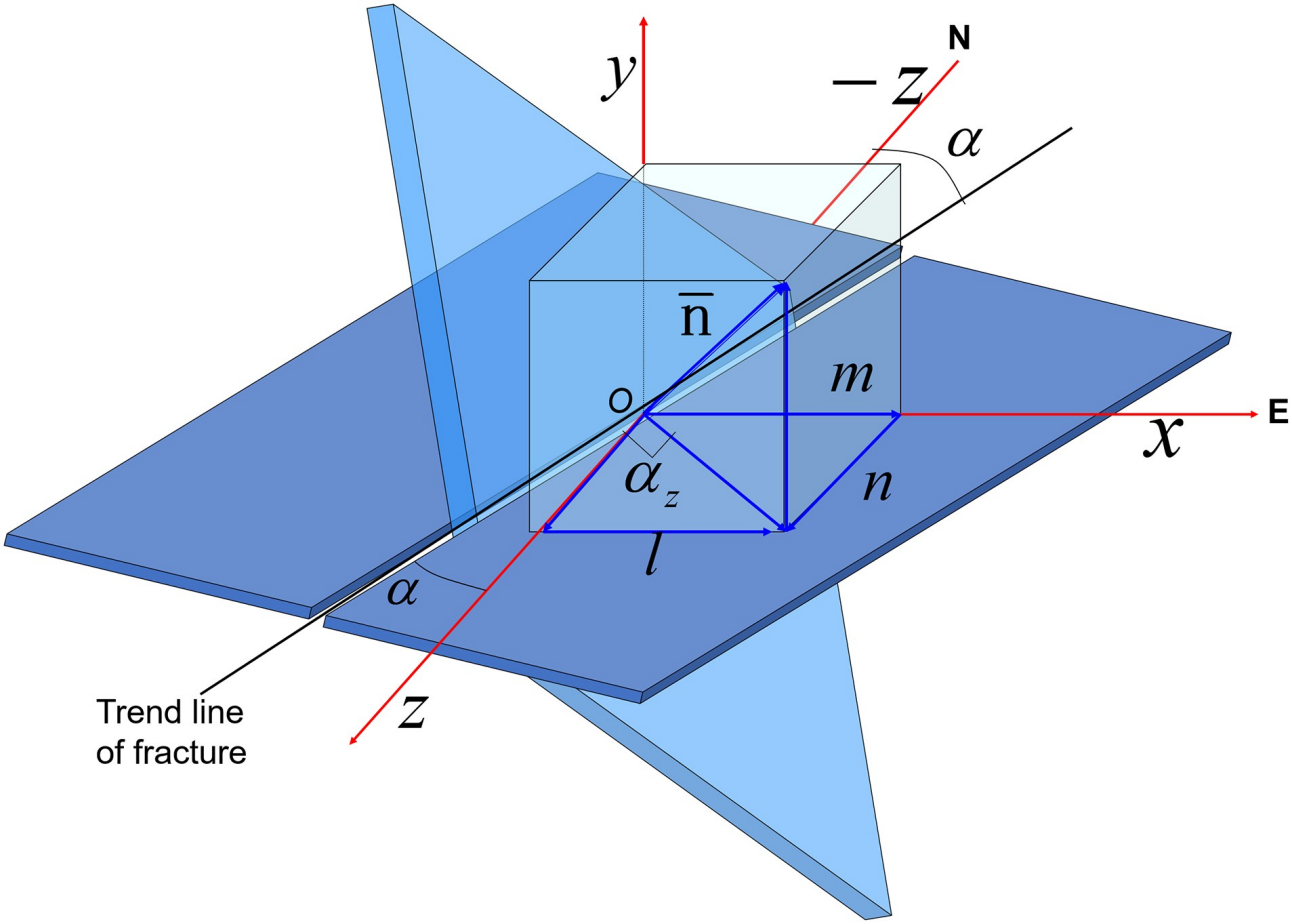
Diagram of the fracture strike and dip angle in the 3-D global coordinate system.

In a 3-D global coordinate system, the calculation of fracture strike and dip depends on a reasonable projection of coordinates, where the *x*-axis aligns with the east-west direction, the *y*-axis aligns with the north-south direction, and the *z*-axis aligns with vertical direction. Thus, the strike angle (*α*) could be characterized in the *x-o-z* plane using the normal vector of its fracture surface. Here $\overset{-}{\text{n}}\;=\;\{l\;m\;n\}$ denoted the cosine of the normal vector projected in the *x-o-z* plane, whose inclination angle with respect to *z-axis* (*α*_*Z*_) was obtained via the following:

$$\begin{array}{ll} \text{if}\;0{}^{\circ}\leq \alpha_{z}<90{}^{\circ}, & \alpha =90{}^{\circ}-\alpha_{z} \end{array}$$ (9)

$$\begin{array}{ll} \text{if}-90{}^{\circ}\leq \alpha_{z}<0{}^{\circ}, & \alpha =\left(-90{}^{\circ}-\alpha_{z}\right)+360{}^{\circ} \end{array}$$ (10)

where *α*_*Z*_ = *arctan*(−*l*/*n*) (Fig 7).

Geologically speaking, in the a 3-D global coordinate system, the inclination angle between the *lx* + *my* + *nz* = 0 plane and *y* = 0 plane (α_*dip*_) was defined as the inclination angle between the fracture surface and *x-y* plane, which was given by:

$$cos\;\alpha_{dip}\;=\;\frac{\left|l\cdot 0+m\cdot 1+n\cdot 0\right|}{\sqrt{l^{2}+m^{2}+n^{2}}\sqrt{0^{2}+1^{2}+0^{2}}}\;=\;\frac{\left|m\right|}{\sqrt{l^{2}+m^{2}+n^{2}}},0{}^{\circ}\leq \alpha_{dip}\leq 90{}^{\circ}.$$ (11)

#### Fracture seepage parameters

Generally, for multiple sets of fractures, the relationship between fracture porosity and fracture volume density and aperture was illustrated as:

$$\varphi_{ft}\;=\;\sum_{i}^{m}b_{i}{D_{vf}}_{i}$$ (12)

where *m* was the number of fractures; *b*_*i*_ was the aperture of group *i* (m); *D*_*vfi*_ was the volume density of group *i* (m^2^/m^3^); *φ*_*ft*_ was the total fracture volume, as decimal type. When the fracture permeability is calculated under the principal stress space, the principal stress value of the space node can be obtained by FE simulation, but the principal stress direction is inconsistent with the coordinate axis direction of global Cartesian coordinate system. Therefore, the mechanical model of fractures permeability cannot be directly applied. For arbitrarily distributed 3-D fractured media regions, according to Kronecker conversion formula [54] and principle of coordinate transformation [55], the permeability of three coordinate axes for a group of parallel fractures in the global coordinate system could be characterized as:

$$\left[\begin{array}{c} k_{fX}\\ k_{fY}\\ k_{fZ} \end{array}\right]\;=\;\frac{b^{3}D_{lf}}{\text{12}}\left[\begin{array}{c} \text{1-}{n_{f1}}^{2}\\ \text{1-}{n_{f2}}^{2}\\ \text{1-}{n_{f3}}^{2} \end{array}\right]\;=\;\frac{b^{3}D_{lf}}{\text{12}}\left[\begin{array}{c} 1-{\left(cos\;\alpha_{11}sin\;\theta +cos\;\alpha_{31}cos\;\theta \right)}^{2}\\ 1-{\left(cos\;\alpha_{12}sin\;\theta +cos\;\alpha_{32}cos\;\theta \right)}^{2}\\ 1-{\left(cos\;\alpha_{13}sin\;\theta +cos\;\alpha_{33}cos\;\theta \right)}^{2} \end{array}\right]$$ (13)

Similarly, if there were multiple groups of fractures, the permeability of the group *i* fractures in the three coordinate axes was:

$$\left[\begin{array}{c} k_{fXi}\\ k_{fYi}\\ k_{fZi} \end{array}\right]\;=\;\frac{{b_{i}}^{3}D_{lfi}}{\text{12}}\left[\begin{array}{c} \text{1-}{n_{f1i}}^{2}\\ \text{1-}{n_{f2i}}^{2}\\ \text{1-}{n_{f3i}}^{2} \end{array}\right]\;=\;\frac{{b_{i}}^{3}D_{lfi}}{\text{12}}\left[\begin{array}{c} 1-{\left(cos\;\alpha_{11i}sin\;\theta_{i}+{cos\;\alpha_{31}}_{i}cos\;\theta_{i}\right)}^{2}\\ 1-{\left({cos\;\alpha_{12}}_{i}sin\;\theta_{i}+cos\;\alpha_{32i}cos\;\theta_{i}\right)}^{2}\\ 1-{\left({cos\;\alpha_{13}}_{i}sin\;\theta_{i}+cos\;\alpha_{33i}cos\;\theta_{i}\right)}^{2} \end{array}\right]$$ (14)

Thus, the total fracture permeability was calculated using Eq (15), namely:

$$\left[\begin{array}{c} {k^{T}}_{fX}\\ {k^{T}}_{fY}\\ {k^{T}}_{fZ} \end{array}\right]\;=\;\sum_{i}^{m}\left[\begin{array}{c} k_{fXi}\\ k_{fYi}\\ k_{fZi} \end{array}\right]\;=\;\sum_{i}^{m}\frac{{b_{i}}^{3}D_{lfi}}{\text{12}}[\begin{array}{c} \text{1-}{n_{f1i}}^{2}\\ \text{1-}{n_{f2i}}^{2}\\ \text{1-}{n_{f3i}}^{2} \end{array}]$$ (15)

where, *m* was the number of fractures; *D*_*lfi*_ was the linear fracture density of group *i* (1/m); *K*_*fXi*_, *K*_*fYi*_a and *K*_*fZi*_ were the permeability in the *x*-axis, *y*-axis and *z*-axis directions, respectively (m^2^); *α*_11*i*_, *α*_12*i*_ and *α*_13*i*_ were the angles between *σ*_1_ and axis-*x*, *y*, *z* for group-*i* fractures (°); *α*_21*i*_, *α*_22*i*_ and *α*_23*i*_ were the angles between *σ*_2_ and axis-*x*, *y*, *z* for group-*i* fractures (°); *α*_31*i*_, *α*_32*i*_ and *α*_33*i*_ were the angles between *σ*_3_ and axis-*x*, *y*, *z* for group-*i* fractures (°); *θ* was the angle between the short axis direction of fracture surface and *σ*_1_, namely the rupture angle of rock (°); *K*^*T*^_*fX*_, *K*^*T*^_*fY*_ and *K*^*T*^_*fZ*_ were the total permeability in the *x*-axis, *y*-axis and *z*-axis directions, respectively (m^2^); (*n*_*f*1*i*_, *n*_*f*2*i*_, *n*_*f*3*i*_) were the three components of unit normal vector of group-*i* fracture surfaces, respectively, which could be calculated by Eq 16:

[nf1nf2nf3]=[cosα11cosα21cosα31cosα12cosα22cosα32cosα13cosα23cosα33][sinθ0cosθ]=[cosα11sinθ+cosα31cosθcosα12sinθ+cosα32cosθcosα13sinθ+cosα33cosθ] (16)

### Fracture aperture under present-day stress

For our modeling purposes, we assumed that the paleostress field generated fractures, while the present-day stress field induces minor changes in fracture size but does not produce new fractures. We made further modification to the existing palaeomechanical models. Considering the influences of the normal stress and shear stress on the aperture of a fracture, Hicks et al. [56] and Jing et al. [57] derived an equation to calculate the aperture of a fracture due to the current in situ stress field as follows:

$$b_{m}\;=\;\frac{b_{0}}{1+\frac{9\sigma_{n}^{\prime }}{\sigma_{nref}}}+b_{res}$$ (17)

where *b*_*0*_ and *b*_*m*_ were the original and current aperture of the fracture (m), respectively; σ*’*_*n*_ was the effective normal stress (MPa), namely was the result of the normal stress acting perpendicular to fracture plane minus the fluid pressure acting inside fracture [8], [9]; *b*_*res*_ was the residual aperture of the fracture (m); and *σ*_*nref*_ was the corresponding effective normal stress (MPa) when the fracture aperture decreased by 90%. As for the parameter *σ*_*nref*_, many researchers [58], [59], [60] have proposed different determination ranges based on their experimental data. Through a series of permeability tests under variable confining pressure, Qin [60] showed that the reasonable value 30 MPa should be applied to low-permeability sandstones with uniaxial compressive strength ranging from 30 to 70 MPa. Because of minimum effects on total seepage capability, or difficult to be determined by conventional means, the parameter *b*_*res*_ could be often ignored in calculations. Additionally, as a critical parameter, the effective stress $\sigma_{n}^{\prime }$ was the result of the normal stress acting perpendicular to fracture plane (compression assumed positive) minus the fluid pressure acting inside fracture [8], [9]: $\sigma_{n}^{\prime }\;=\;\sigma_{n}-P$. Therefore, the equation for calculating fracture porosity under present geostress field is modified as:

$$\varphi_{f}\;=\;b_{m}D_{vf}$$ (18)

Generally, the higher the filling degree in a fracture, the lower its aperture and porosity are expected to be. Here we introduced a novel method of mineral filling coefficient to characterize the filling degree in fractures form the perspective of natural hydraulic fracturing. In detail, under two limit conditions that when a fracture was most or full filled by minerals (calcite, quartz, gypsum or mud) the filling coefficient *C* was equal to 1, and when the fracture was almost unfilled, the *C* was zero. When the fracture was approximately half or more than filled, the *C* was assigned the value 0.5, but when the deposited minerals accounted for a quarter of the fracture volume, the *C* might be assigned 0.25 as the estimator. Therefore, this leads to two simpler expressions for estimating the effective fracture aperture and porosity relative to filling degree required for opening-mode fracturing:

$$b_{fe}\;=\;(1-C)b_{m}$$ (19)

$$\varphi_{ef}\;=\;(1-C)\varphi_{f}$$ (20)

where *b*_*fe*_ and *ϕ*_*ef*_ were the effective fracture aperture and porosity, respectively; *b*_*m*_ was the original fracture aperture before fillings occurred.

In this way, the calculation formula of effective fracture permeability under the current stress field is modified to:

$$\left[\begin{array}{c} k_{\text{fXe}}\\ k_{\text{fYe}}\\ k_{\text{fZe}} \end{array}\right]\;=\;(1-C{)}^{3}\left[\begin{array}{c} k_{\text{fX}}\\ k_{\text{fY}}\\ k_{\text{fZ}} \end{array}\right]\;=\;\frac{(1-C{)}^{3}b_{m}^{3}D_{\text{lf}}}{12}\left[\begin{array}{c} 1-{\left(cos\;\alpha_{11}sin\;\theta +cos\;\alpha_{31}cos\;\theta \right)}^{2}\\ 1-{\left(cos\;\alpha_{12}sin\;\theta +cos\;\alpha_{32}cos\;\theta \right)}^{2}\\ 1-{\left(cos\;\alpha_{13}sin\;\theta +cos\;\alpha_{33}cos\;\theta \right)}^{2} \end{array}\right]$$ (21)

## Discussion

### Geomechanical simulation of tectonic stress field

**Fig 8:**
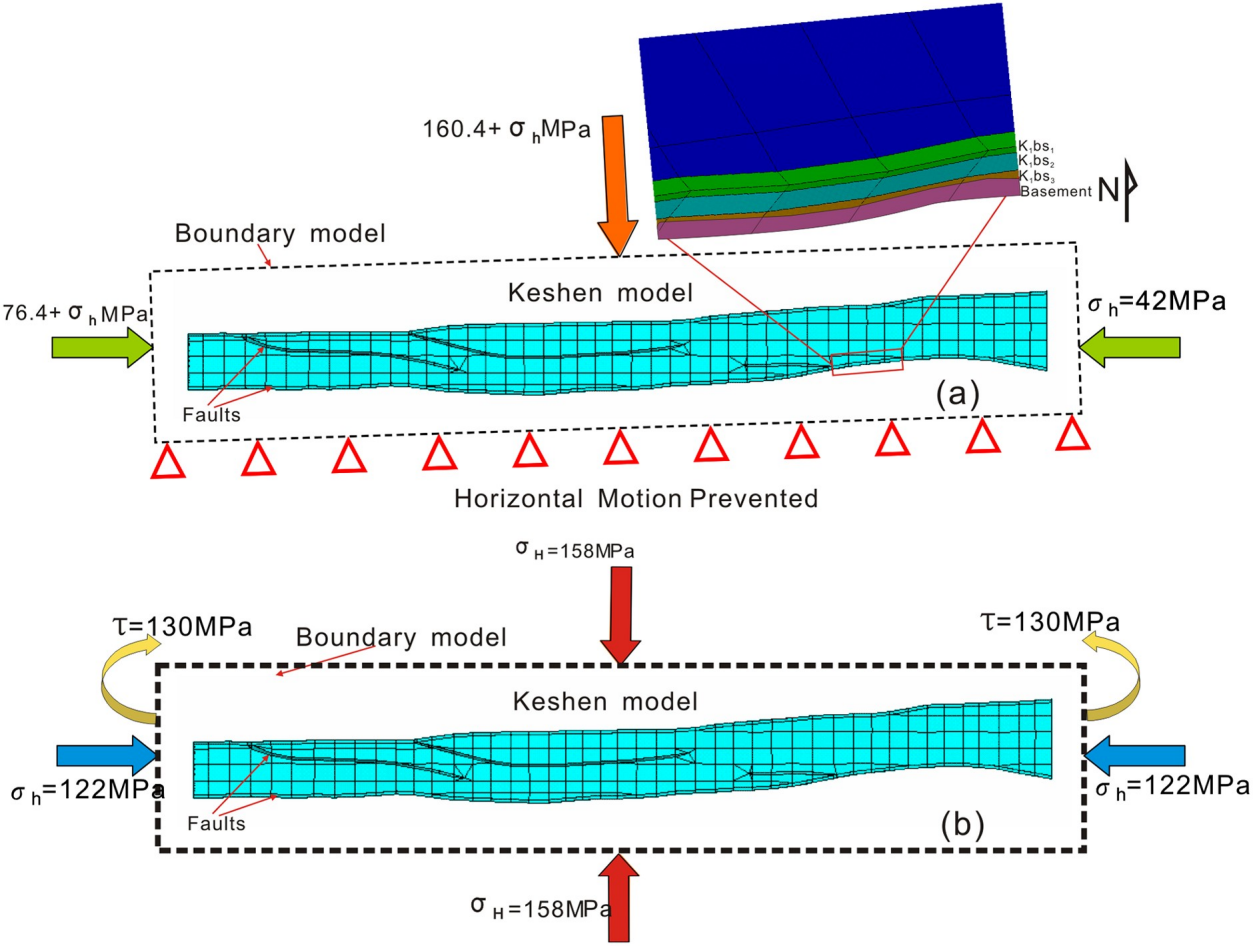
Simplified nested models and meshing of the initial 3-D FE models of the Bashenjiqike Formation in the Keshen gas field. (a) boundary conditions of a palaeostress field model; (b) boundary conditions of a current stress field model.

Geomechanical modeling with Finite Element Method (FEM) was conducted to simulate the paleostress and current stress distributions for prediction of fracture development and distribution. The general-purpose finite element code ANSYS (15.0, ANSYS, Inc., USA) was selected for this study because it is well-suited for analyzing geomechanical problems over a wide range of scales in one, two, and three dimensions [61], [23], [62], [27]. The initial three-dimensional model geometries were constructed for the restored geologic area constrained by field measurements during Pliocene Kuqa Period [42] and incorporated the generalized mechanical stratigraphy of the Bashijiqike Formation (K_1_ *bs*_*1*_, K_1_ *bs*_*2*_ and K_1_ *bs*_*3*_). The average thickness of the K_1_ *bs*_*1*_, K_1_ *bs*_*2*_ and K_1_ *bs*_*3*_ member within the FE model was set to be 50 m, 180 m and 65 m, respectively. The target zone associated with the upper cover layers (i.e. *K*, *E*, *N* Formations) and basement (i.e. *J* Formation) were considered as an isolated body or boundary conditions for simulation (Fig 8a). All model layers were discretized using primarily three-node triangle plane strain elements along with some four-node quadrilateral elements (311,226 total elements in the model).

Based on regional analysis and acoustic emission of rocks, the Kuqa depression has primarily experienced three important thrusting movements during the Himalayan Period and Neotectonics Period, which can be defined as Early Initial Compressional Stage, Mid-term Strong Thrusting and Uplift Stage with greater slip displacement and Late Uplift and Recoil Stage with higher fold amplitude, respectively [23], [21], [43], [63]. It was important that the Kuqa tectonic movement after Pliocene sedimentary was the crucial time of fracture generation in the Keshen reservoir, and the direction of maximum tectonic stress in the period was identified as nearly NNW352° [21], [63], with a magnitude of about 370 MPa. Then, based on paleotectonic deformation, a methodology that included both inverse and forward methods was used to identify the magnitude of minimum paleostress field, which was about 70 MPa. Considering the Kuqa period after Pliocene sedimentary as the crucial time of fracture formation in the Bashijiqike reservoir, the cover layers are set to approximately 5500 m thick. Consequently, in the first step, we selected the southern edge (hinterland side) as the fixed boundary in the simulated stratigraphy so that the Keshen anticline could uplift if the stress state was appropriate (Fig 8a). The second step imposed a deformation of fault-related fold by forming a displacement boundary condition along the north (foreland) side of the model. The base of the model was pinned, a gravity load was applied to the entire model domain, and the system was allowed to reach equilibrium.

The state of in-situ stress is described by a stress tensor, which comprises three orthogonal principal stresses, each with an orientation and magnitude [44], [64], [65]. In this study, XMAC logging, acoustic emission test, hydraulic fracturing test and differential deformation analysis were used to determine the magnitude of maximum horizontal stress (*σ*_*H*_), minimum horizontal stress (*σ*_*h*_) and vertical stress (*σ*_*v*_) of drilling wells in the Keshen area (Table 5). The horizontal stress orientation in the study area was determined from paleomagnetic correction, wave velocity anisotropy test, and drilling-induced fractures (DIF). As shown in Table 6, the results indicated that the orientation of maximum horizontal stress in wells had obvious zoning characteristics, mainly ranging from NW322° to NE 51°. Interestingly, the direction of *σ*_H_ in the central region was nearly north-south direction, distinctly different from the that of western and eastern regions. From Table 5 we could see that the maximum horizontal stress values at different depths of the study stratum were significantly higher than that of vertical stress, which indicated that a Type III stress environment (i.e., *σ*_*H*_>*σ*_*v*_>*σ*_*h*_) was widely developed in Keshen area at present. The study area is primarily affected by the southern Indian plate and northern Kazakhstan-Junggar plate, with approximately twice the force as that from the North China plate [66]. After several design improvements and repeated tests, the reasonable scheme for mechanical boundary condition (including stress and displacement constraints) were finally confirmed. A magnitude of 122 MPa was applied to the E90° and W270° boundaries of the nested model, respectively, as horizontal *σ*_*h*_ (Fig 8b). Simultaneously, A magnitude of 158 MPa was applied to the N360° and S180° boundaries of the nested model, respectively, as horizontal *σ*_*H*_. The vertical stress could be calculated and applied automatically in software based on gravitational acceleration. As for the variations of stress orientation with the slipping of boundary faults, we should take into account the extra shear stresses while applying loading conditions. In detail, we applied dextral and sinistral shearing stresses to the west and east boundaries, respectively, both with a magnitude of 130 MP.

**Table 5:** Orientation and magnitude of three current principal stresses in Keshen area.

Well	Depth (m)	Mean *σ*_*H*_ value (MPa)	Mean *σ*_*H*_ azimuth (MPa)	Mean *σ*_*h*_ value (MPa)	Mean *σ*_*v*_ value (MPa)	Types of stresses
**A1-1**	6925–7180	164	341°	123	154	III
**A1-3**	6888–7180	163	336°	122	153	III
**A3-1**	6868–6885	164	352°	121	154	III
**A2-1**	6486–6793	156	18°	110	148	III
**A2-2**	6650–6995	164	35°	120	150	III

**Table 6:** Material properties used in the finite element Keshen reservoir geomechanical model. The values are the weighted averages of the variables measured during the mechanical experiments on drilling cores.

Layers	ρ (g/cm^3^)	*E* (GPa)	μ	Internal friction angle (°)	Cohesion (MPa)
**K**_**1**_ ***bs***_***1***_	2.64	4.73	0.15	49.27	5.46
**K**_**1**_ ***bs***_***2***_	2.63	4.73	0.15	53.63	5.78
**K**_**1**_ ***bs***_***3***_	2.60	6.22	0.12	51.14	6.21
**Boundary rock**	2.64	4.70	0.16	51.72	4.31
**Faults**	2.40	4.50	0.20	63.10	3.65

To date, there have been hardly any published anisotropic models of the paleo-stress field of compressional basins, no far any other stress evolution models [67], [68]. Here our study was focused on the paleo-folding stress field, as opposed to building a detailed and complicated model for the present-day field. For simplicity, the mechanical parameters density of model, such as Young’s modulus, and Poisson’s ratio variables were set to the same value for each layer, respectively. Based on the above mechanical experiments, the material properties (i.e., density, Young’s modulus, Poisson’s ratio, friction angle, and cohesion) were assigned to the elements representing the various lithologies (Table 6). The processing method used to determine the material properties of fault zones is important to the outcome of the geomechanical modeling because it directly influences the results of numerical simulation, for instance, the distributions of stresses and fractures [27]. Generally, the Young’s modulus of fault zone is just 65–85% of the elastic modulus of a corresponding normal stratum [27], [28], which indicates that the strength parameters of fault zone is obviously lower than that of intact rocks in tight sandstone areas.

### Prediction of fracture distribution

According to above deduced geomechanical models with respect to the composite rock failure criterions, the initial fracture parameters (density, aperture porosity and permeability) related to stress, strain and strain energy density can be calculated or directly extracted from the numerical simulations of paleostress field during the Kuqa tectonic movement of the Late Himalayan stage. Based on the simulated ancient fracture parameters and considering the impact of current stress field on fracture properties, we could predict the spatial distributions of fracture aperture and porosity for each element/layer, which were displayed on Ansys platform. As for the spatial filling coefficients of fracture in modeling process, one effective method was to use cokriging which considered the paleofluid migration direction to interpolate the filling degree of different layers in the whole region, which was primarily constrained by well-point data.

**Fig 9:**
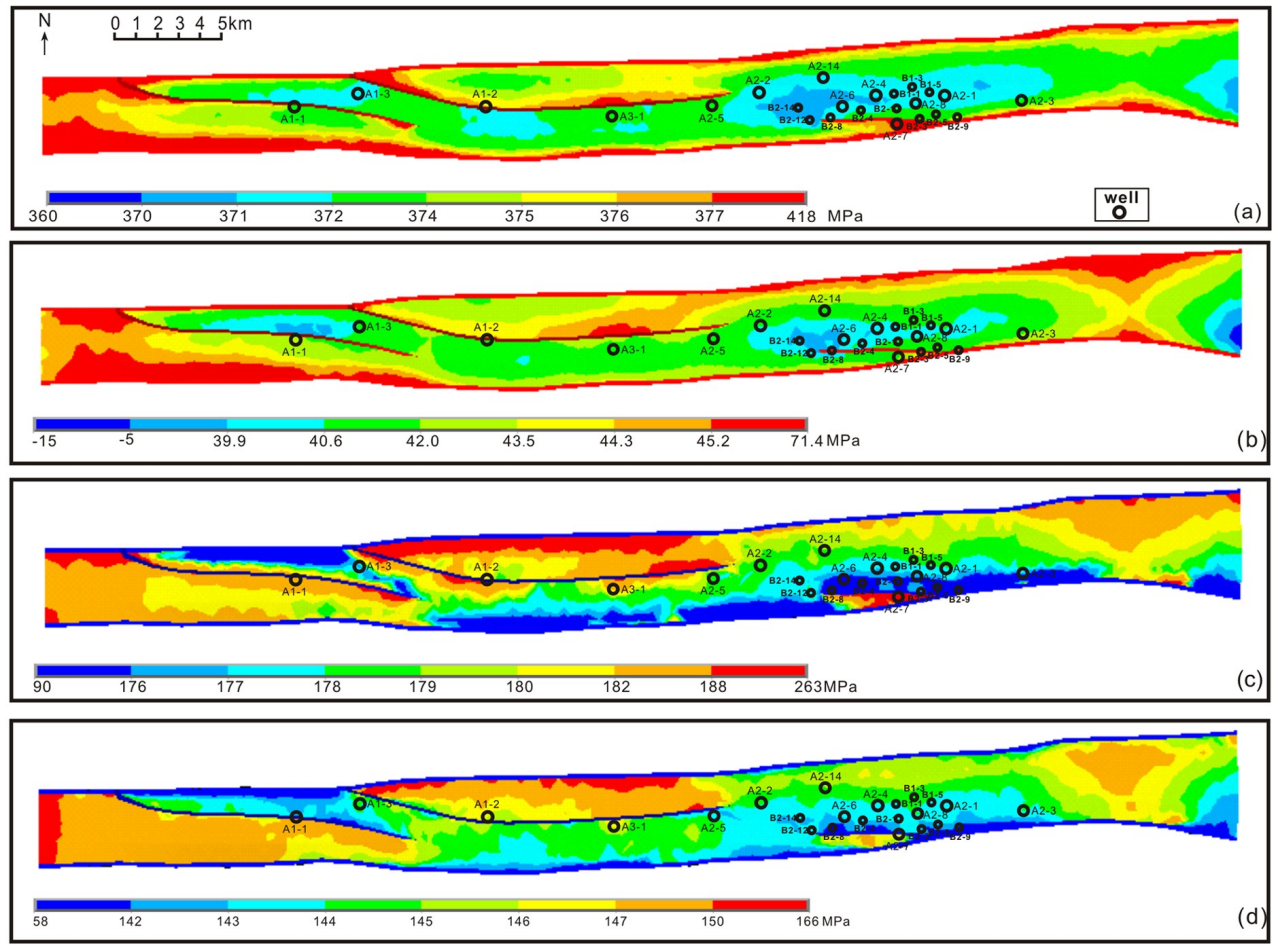
The distribution maps of paleotectonic and current stress field in Bashenjiqike Formation. (a) Maximum principal palaeostress of K_1_ *bs*_*2*_; (b) Minimum principal palaeostress of K_1_ *bs*_*2*_; (c) Maximum current principal stress of K_1_ *bs*_*2*_; (d) Minimum current principal stress of K_1_ *bs*_*2*_.

In this paper, compressional stress was positive and tensile stress was negative. In the K_1_ *bs*, the maximum principal stress (σ_1_), indicative of compression, ranged from 360 MPa to 418 MPa (Fig 9a). The minimum principal stress (σ_3_), indicative of both compression and tension, mainly ranged from -5 MPa to 71.4 MPa (Fig 9b). Fig 9b showed that weak tensile stress (i.e., negative values) was only distributed in the top of Keshen anticline, and consistent with the long axis of fault-related fold, indicating the most probable development zones of tension fractures. The distributions of three principal stresses were similar to each other, all of them were primarily fault-controlled and secondly fold-controlled. For example, after rapid energy releasing in the rock, the fault zone showed lower stress values, and a transformation of stress field appeared near major faults, such as the higher stress values in the footwall and lower stress values in the hanging wall. Similarly, in the simulated current stress field, the distributions of maximum horizontal stress (*σ*_*H*_), minimum horizontal stress (*σ*_*h*_), and vertical stress (*σ*_*v*_) were similar, and the gravity and boundary stresses played the major role within Keshen area (Fig 9c and 9d). The maximum horizontal stress was horizontal, with values varying between 90 MPa and 263 MPa, indicative of compression. Relative lower *σ*_*H*_ values mainly were located in the middle part of the gas field, and gradually increased to both east and west directions. Whereas the *σ*_*h*_ values decreased from north to south in the gas field, which indicated that the stress concentration was primarily come from the resistance of southern plate.

**Fig 10:**
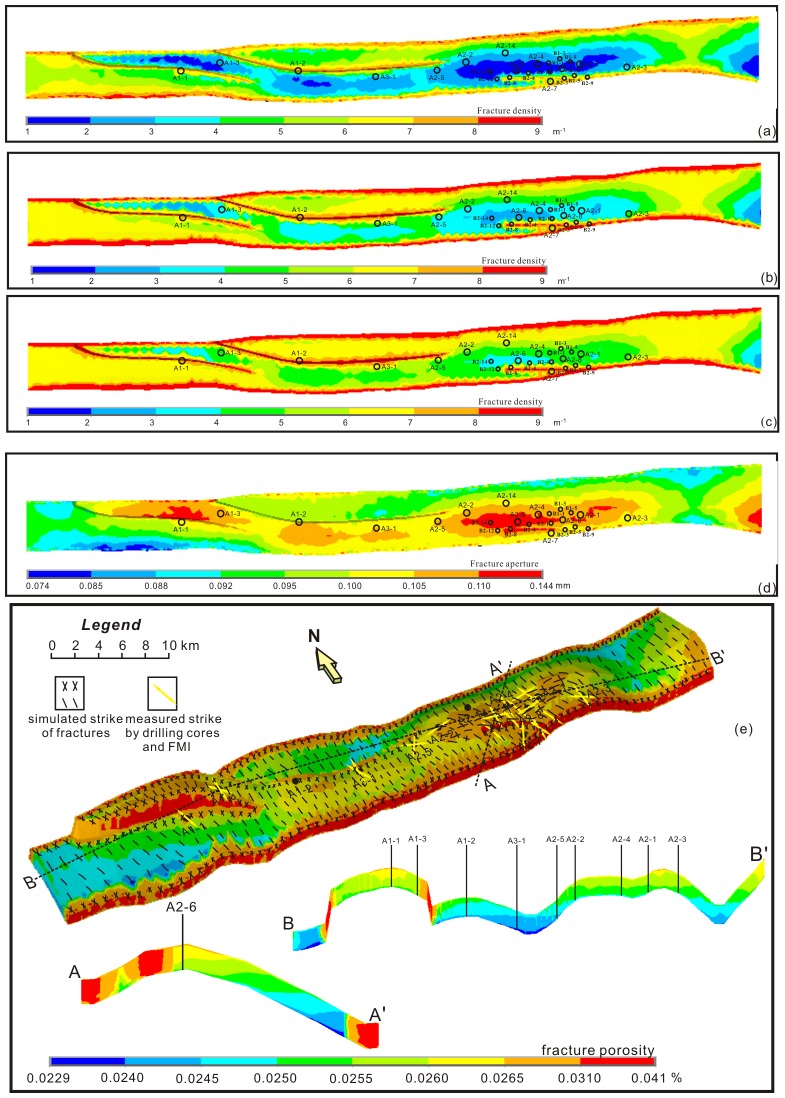
The distribution maps and sections of fracture parameters in Bashenjiqike Formation. (a) simulated fracture density of K_1_ *bs*_*1*_; (b) simulated fracture density of K_1_ *bs*_*2*_; (c) simulated fracture density of K_1_ *bs*_*3*_; (d) simulated current fracture aperture of K_1_ *bs*_*1*_; (e) simulated current fracture porosity and fracture strike of K_1_ *bs*, including two sections in directions of SW-NE and E-W.

After superposition of paleostress field and current stress field in Keshen area, the current simulated linear fracture density ranged mainly from 1 to 9 m^-1^ in the plane (Fig 10a, 10b and 10c). Horizontally, areas with well-developed tectonic fractures were mainly located in fold limbs, fault zones (such as the Wells A2-7, B2-3, B2-8 and B2-9), locations of changes in the orientation of faults, zones on footwall (such as the Wells A1-1, A1-2), and southern regions around front limb. Vertically, the bottom layer had a relative higher density than the top layer, such as fracture linear density of K_1_ *bs*_*3*_ is higher than that of K_1_ *bs*_*2*_ and K_1_ *bs*_*1*_, indicating that it was highly fractured and more brittle. Additionally, as a comparison, the high-value areas of current fracture aperture were roughly counter to the areas with higher fracture linear density, such as on top of the fold fracture aperture was high, but the fracture density was low, and in northern limb of fold the fracture density was high, but the fracture aperture was low (Fig 10d). Vertically, the current fracture aperture decreased with increasing strata depth, which was just opposite to the distribution of fracture density. However, the predicted well-developed and relatively well-developed areas of fracture density coincided with the areas with high fracture aperture, including the eastern fault zones near the Wells A2-6 and A2-7.

As an important factor in determining the quality and yield of tight gas reservoirs, the distribution of current fracture porosity was very similar with that of fracture aperture. As shown in Fig 10e, the most well-developed porosity zones were mainly located in (1) the northwest, southeast, and middle of the study area; (2) around anticlines; (3) around faults; and (4) the upper thin sandstones. In the anticline area, vertically, fracture porosity gradually decreased from the anticline core to the wings. Comparison of different-scale faults showed that the influence of faults at the high point on fracture porosity was more significant than that in lower position. In fractures associated with faults at the lower position, core observations and other evidence suggested that cement might be easily deposited simultaneously with fracture opening and propagation, partially filling and decreasing the large aperture parts [14]. As illustrated in Fig 10e, the fractures simulated in the K_1_ *bs* can be divided into four sets, namely set I (NW-SE), Set II (NNE-SSW), set III (NE-SW) and set IV (nearly EW), where the former two sets were predominant, which basically coincided with orientations in drilling cores and FMI. Set I fracture strike 295°, oblique to the fold axial trend and boundary faults with acute angle, and were interpreted as a regional fracture set that was present before the Keshen anticline and with the stress field of sinistral rotation and compressive shearing. Set II fractures striking 20° and set III fractures striking 65° near fault zones, were interpreted as composite conjugate fracture set associated with faulting related to the NNW-oriented Himalayan compression during initial anticline growth. Set IV fractures striking 96°, nearly parallel to the fold trend, were found mainly within the crest and were interpreted to have formed in response to local tensile stresses during folding.

**Fig 11:**
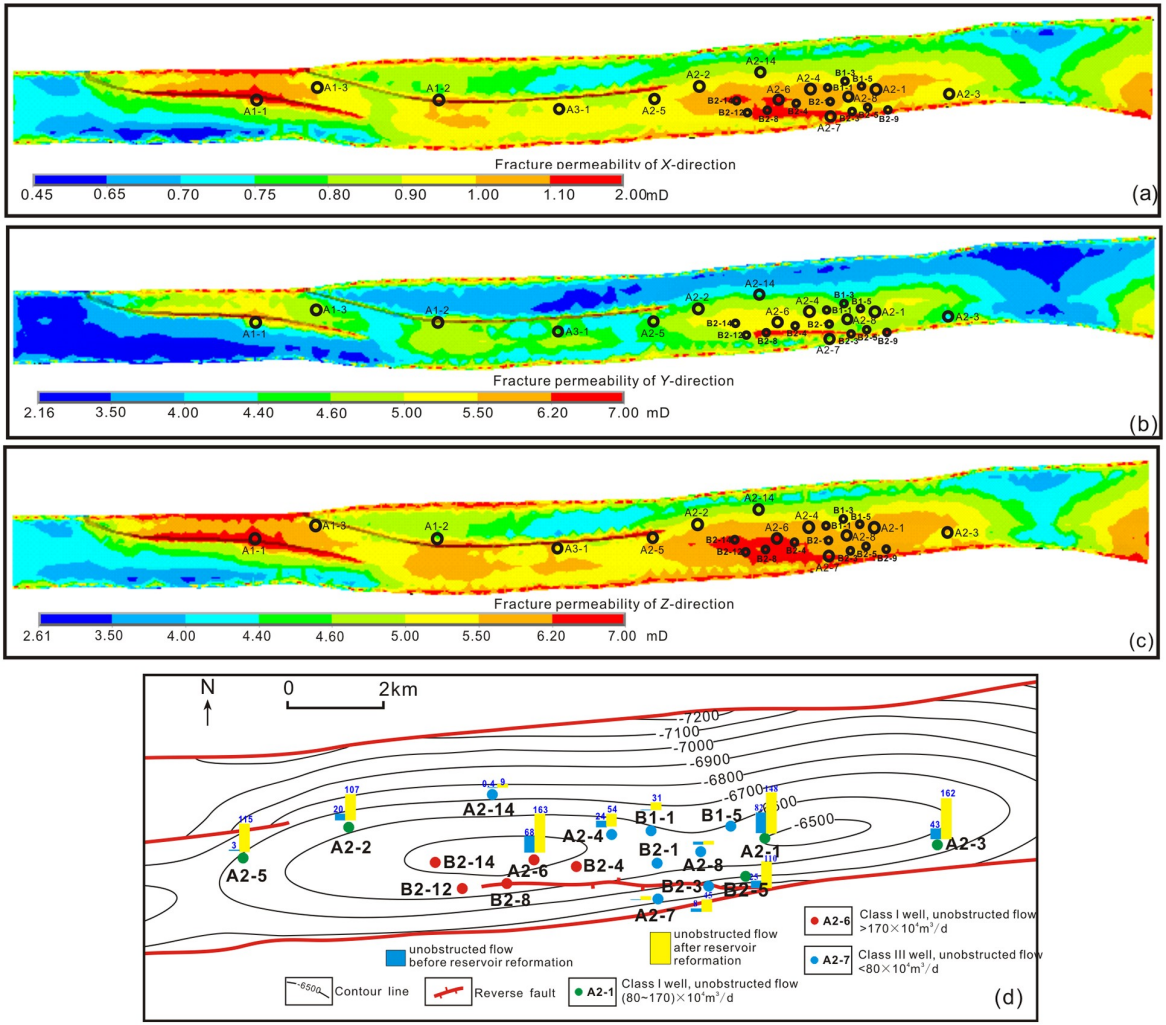
The distribution maps of effective fracture permeability in K_1_ *bs*_*2*_. (a) simulated fracture permeability in east-west direction; (b) simulated fracture permeability in south-north direction; (c) simulated fracture permeability in vertical direction; (d) distribution map of single-well unobstructed flow.

In general, the high permeability zone in Keshen area was concentrated in the high structure and fault zone, and the permeability value decreased with depth (Fig 11a, 11b and 11c). At the same time, the permeability of fractures in different directions was mainly related to the occurrence of fractures. The fractures in the study area were dominated by vertical joints, so the permeability value in the vertical direction was the highest. The main trend of fractures in the study area was nearly S-N direction, so the permeability in the north-south direction was obviously higher than the east-west direction. The cause of this change in fracture permeability could be briefly explained by stress state transitions (Fig 9). In the shallow part of Keshen anticline, the maximum principal stress was north-south direction, the minimum principal stress was vertical, and the intermediate principal stress was east-west. Under this condition, the permeability had the characteristics that the east-west direction was greater than the north-south direction, and the north-south direction was greater than the vertical direction. In the deep part, after reaching a certain depth, the stress field changed, the vertical stress increased to the intermediate principal stress, and the minimum principal stress was the east-west direction. It was observed that it was only necessary to rotate the shallow geostress model 90° along the σ_1_ axis to obtain the deep-ground stress model, and the permeability in the corresponding direction also changed. As a result, the permeability in the corresponding direction also changed, the maximum permeability appeared in the vertical direction, the minimum permeability became east-west, and the north-south permeability was centered.

As shown in Table 7, the overall geomechanical FE modeling results at the wells were in agreement with in-situ core observations and formation micro-imaging (FMI). The average fracture density at Wells A2-7 and B2-8 in *K*_*1*_ *bs* was as high as 9.36/m and 5.62/m, respectively, according to the core and FMI in-situ measurements, while modeling results yielded 9.04/m and 6.18/m, for the same two wells, respectively. Comparison diagram of all 12 sample spots in the Bashijiqike Formation indicated little error between the computed and in-situ results. Only a few samples showed relative errors in average linear fracture density greater than 20% and these larger relative errors were distributed along fault ending, such as Wells B2-9, A2-5 and A2-8. Compared with the traditional core observation method, important in-situ physical parameters (i.e., aperture and porosity) of fractures are more easily obtained by FMI interpretation and CT scanning accurately and continuously.

**Table 7:** The Comparison between predicted results and measured results and error analysis in the Keshen reservoir.

Wells	Fracture density (m^-1^)		Fracture aperture (mm)		Fracture porosity (%)		Data sources
	Measured results	Simulated results	Measured results	Simulated results	Measured results	Simulated results	
A2-1	1.49	1.37	0.095	0.102	0.026	0.0257	FMI
A2-2	2.60	2.24	0.083	0.096	0.0233	0.0245	FMI
A2-4	3.80	3.96	0.124	0.101	0.037	0.0261	FMI
A2-5	2.23	2.92	0.081	0.094	0.0373	0.0271	CT, Cores
A2-6	1.39	1.32	NA	0.129	NA	0.042	FMI
A2-7	9.04	9.36	0.083	0.105	0.041	0.046	FMI, CT, Cores
A2-8	2.90	2.11	0.066	0.097	0.062	0.054	FMI
A3-1	5.51	5.32	0.087	0.097	0.029	0.0261	FMI
B2-3	4.25	3.90	0.143	0.148	0.059	0.050	CT, Cores
B2-4	1.82	1.80	0.134	0.146	0.038	0.032	CT, Cores
B2-5	8.15	8.44	0.101	0.114	0.0487	0.051	CT, Cores
B2-8	6.18	5.62	0.142	0.153	0.069	0.054	CT, Cores
B2-9	4.88	3.55	NA	0.136	NA	0.044	Cores
Correlation coefficient	*R*^2^ = 0.959		*R*^2^ = 0.793		*R*^2^ = 0.826		
Average error	11.20%		15.28%		14.16%		

It is worthwhile to note that in Table 7, the average relative error between the predicted fracture apertures and the measured fracture apertures in was not more than 20%, except for one well exceeding 35% (S3 Appendix). Similarly, based on data analysis, the majority of predicted fracture porosities had nice fit with the measured fracture porosities (average relative error<20%), indicating that the present prediction of tectonic fractures by geomechanical superposition method in the brittle tight sandstone regions was believable. As for the existing larger errors, it was inferred that probably the stress concentration phenomena induced by lithology variation or fault zone structure were still hard to be correctly reflected by normal FE mesh partition, which directly led to the uncertainty of simulation results including the development and distribution of tectonic fractures. As shown in Fig 11d, in the eastern part of the Keshen anticline, although there was large difference between the unobstructed flow before and after the reservoir reformation, the distribution trend of the latter was basically consistent with the fracture permeability distributions.

## Conclusions

An adapted geomechanical method was used to calculate the multiple parameters of fracture governing the development of the tight sandstone reservoirs for unconventional resources in the Kuqa Depression within Tarim Basin, China. This method was selected for its superior applicability in developed fracture areas after key tectonic movement and its adaptability to fit the FE modeling. Its predictions also agreed well with present in-situ core observations and FMI interpretation.

From the rock mechanics experiments under continuous loading, fracture evolution of tight sandstone (sometimes containing a small number of micro-fractures) was summarized into three stages: (1) initial compaction stage, (2) propagation stage, and (3) coalescence stage extracted from three-dimensional CT scan. When reaching peak shear strength, tight sandstone rocks experienced volume expansion and micro-fractures rapidly connect to each other. This value tended to be τ = 0.85 σ_c_ (σ_c_ < 200 MPa), which was the critical sliding value mentioned in Byerlee’s law [69]. From a microcosmic point of view, there existed only three failure modes in brittle rocks: shear fracturing, tensional fracturing, and composite fracturing, which depended mainly on confining pressure, principal stress intensity, and the pre-existing plane of weakness. After many empirical experiments, a relationship between the fracture volume density and stress-strain of tight sandstone reservoirs was finally established. To this end, considering the superimposed effect of paleostress and current stresses, the expressions or geomechanical models were obtained for characterizing the ancient fracture parameters after key tectonic movement and present-day fracture property parameters under different stress states. These expressions/models could be incorporated into a program running on a FE simulation platform, which had become a popular and effective modeling approach in the prediction of fracture generation and spatial distribution. The geomechanical model was, however, limited to the strength prediction for fractured anisotropic rocks and triaxial testing conditions. Further study was suggested to extend the composite modified criterion for anisotropic rock masses and polyaxial testing conditions, with emphasis on the effect of heterogeneous pre-existing fractures.

## Supporting information

S1 Appendix Stress-strain data of samples C1-C4. (DOC)

S2 Appendix CT data of stress and fracture parameters of C3 and C5. (XLSX)

S3 Appendix Measured and simulated fracture data for all wells. (XLSX)
