# Supplementary material for: An improved geomechanical model for the prediction of fracture generation and distribution in brittle reservoirs
Source: PLoS One. 2018 Nov 7;13(11):e0205958. doi: 10.1371/journal.pone.0205958 (PMC6221284; doi:10.1371/journal.pone.0205958)
Supplement: S1 Appendix — (DOC) [file pone.0205958.s001.doc]

**Table. Stress - strain data sheet of Sample C1 under uniaxial compression**

| Time（s） | Axial strain  $\varepsilon_{h}$（$\times10^{-4}$） | Radial Strain$\varepsilon_{r}$（$\times10^{-4}$） | Axial stress  $\sigma_{h}$（MPa） | Time（s） | Axial strain  $\varepsilon_{h}$（$\times10^{-4}$） | Radial Strain$\varepsilon_{r}$（$\times10^{-4}$） | Axial stress  $\sigma_{h}$（MPa） |
| --- | --- | --- | --- | --- | --- | --- | --- |
| 0.00 | 0.00 | 0.00 | 0.00 | 186.44 | 36.92 | -1.36 | 14.74 |
| 10.34 | 1.12 | -0.14 | 1.99 | 189.90 | 37.62 | -1.56 | 15.34 |
| 28.68 | 4.43 | -0.32 | 2.39 | 193.78 | 38.38 | -1.74 | 15.54 |
| 29.80 | 5.11 | -0.42 | 2.79 | 197.18 | 39.08 | -1.99 | 16.34 |
| 32.52 | 5.83 | -0.36 | 2.59 | 200.70 | 39.77 | -2.17 | 16.93 |
| 39.04 | 6.61 | -0.32 | 2.79 | 204.04 | 40.44 | -2.39 | 17.33 |
| 60.18 | 11.13 | -0.31 | 3.19 | 207.82 | 41.18 | -2.62 | 17.93 |
| 62.22 | 12.02 | -0.18 | 3.79 | 211.12 | 41.83 | -2.84 | 18.33 |
| 66.26 | 13.15 | -0.42 | 3.98 | 214.50 | 42.51 | -3.08 | 18.93 |
| 73.60 | 14.58 | -1.00 | 4.18 | 217.56 | 43.10 | -3.26 | 19.32 |
| 80.26 | 15.90 | -1.43 | 4.58 | 222.02 | 43.98 | -3.66 | 20.32 |
| 87.74 | 17.40 | -0.05 | 5.18 | 224.80 | 44.53 | -3.85 | 20.52 |
| 91.32 | 18.10 | -0.27 | 5.38 | 228.22 | 45.21 | -4.11 | 21.12 |
| 98.22 | 19.47 | -0.52 | 5.78 | 231.38 | 45.84 | -4.40 | 21.52 |
| 101.90 | 20.19 | -0.58 | 6.18 | 235.06 | 46.55 | -4.71 | 22.51 |
| 105.82 | 20.97 | -0.72 | 6.37 | 238.44 | 47.22 | -5.05 | 22.71 |
| 109.42 | 21.69 | -0.79 | 6.57 | 241.84 | 47.91 | -5.34 | 23.31 |
| 112.84 | 22.36 | -0.07 | 6.77 | 245.14 | 48.55 | -5.70 | 23.91 |
| 116.62 | 23.12 | -0.16 | 7.17 | 248.74 | 49.27 | -6.17 | 24.11 |
| 120.30 | 23.84 | -0.16 | 7.57 | 252.06 | 49.92 | -6.53 | 24.90 |
| 124.08 | 24.59 | -0.25 | 7.97 | 255.34 | 50.56 | -6.97 | 25.30 |
| 127.84 | 25.34 | -0.27 | 8.37 | 258.62 | 51.23 | -7.46 | 25.70 |
| 132.10 | 26.17 | -0.34 | 8.57 | 262.34 | 51.96 | -8.02 | 26.70 |
| 135.96 | 26.95 | -0.36 | 8.77 | 265.92 | 52.68 | -8.63 | 27.10 |
| 139.84 | 27.72 | -0.38 | 9.36 | 269.54 | 53.39 | -9.20 | 27.29 |
| 143.78 | 28.49 | -0.38 | 9.56 | 273.06 | 54.07 | -9.86 | 28.09 |
| 155.52 | 30.82 | -0.11 | 10.76 | 276.88 | 54.85 | -10.73 | 28.49 |
| 159.72 | 31.63 | -0.22 | 10.96 | 280.40 | 55.55 | -11.57 | 28.89 |
| 163.56 | 32.40 | -0.36 | 11.55 | 284.30 | 56.31 | -12.58 | 29.29 |
| 167.44 | 33.17 | -0.51 | 12.15 | 287.80 | 57.01 | -13.74 | 29.68 |
| 171.34 | 33.95 | -0.67 | 12.55 | 292.00 | 57.82 | -15.37 | 30.08 |
| 174.92 | 34.65 | -0.89 | 13.15 | 296.08 | 58.66 | -17.47 | 30.28 |
| 179.04 | 35.47 | -1.00 | 13.94 | 300.74 | 59.60 | -21.27 | 29.88 |
| 182.72 | 36.20 | -1.21 | 14.14 | 303.52 | 60.20 | -32.49 | 26.30 |
| $\sigma_{c}=P_{\max}/S$ $E=\sigma_{h(50)}/\varepsilon_{h(50)}$ $\mu=-\varepsilon_{r(50)}\varepsilon_{h(50)}$ | | | | | | | |

Table. Stress - strain data sheet of Sample C2 under uniaxial compression

| Time（s） | Axial strain  $\varepsilon_{h}$（$\times10^{-4}$） | Radial Strain$\varepsilon_{r}$（$\times10^{-4}$） | Axial stress  $\sigma_{h}$（MPa） | Time（s） | Axial strain  $\varepsilon_{h}$（$\times10^{-4}$） | Radial Strain$\varepsilon_{r}$（$\times10^{-4}$） | Axial stress  $\sigma_{h}$（MPa） |
| --- | --- | --- | --- | --- | --- | --- | --- |
| 0.00 | -0.01 | -0.04 | 0.20 | 237.98 | 33.74 | -1.34 | 19.04 |
| 13.92 | 0.03 | -0.04 | 2.14 | 241.50 | 34.44 | -1.41 | 20.01 |
| 76.80 | 2.56 | -0.18 | 2.72 | 244.96 | 35.13 | -1.57 | 20.40 |
| 107.24 | 7.91 | -0.69 | 3.50 | 248.68 | 35.86 | -1.75 | 21.37 |
| 118.56 | 10.09 | -0.09 | 3.89 | 252.16 | 36.56 | -1.98 | 22.15 |
| 125.20 | 11.39 | -0.19 | 4.27 | 255.56 | 37.23 | -2.12 | 22.93 |
| 128.28 | 12.01 | -0.23 | 4.47 | 258.98 | 37.89 | -2.21 | 23.32 |
| 131.38 | 12.64 | -0.36 | 5.25 | 263.12 | 38.74 | -2.43 | 24.29 |
| 140.66 | 14.47 | -0.57 | 5.83 | 266.32 | 39.35 | -2.61 | 24.87 |
| 146.90 | 15.70 | -0.73 | 6.22 | 269.94 | 40.07 | -2.78 | 25.84 |
| 150.46 | 16.40 | -0.79 | 6.80 | 311.16 | 40.91 | -2.82 | 26.03 |
| 153.54 | 17.02 | -0.79 | 7.19 | 360.58 | 41.89 | -2.87 | 27.01 |
| 162.66 | 18.82 | -0.02 | 7.97 | 402.86 | 42.73 | -3.00 | 27.78 |
| 165.58 | 19.40 | -0.04 | 8.55 | 442.98 | 43.53 | -3.09 | 28.95 |
| 168.62 | 20.01 | -0.02 | 9.13 | 482.20 | 44.31 | -3.21 | 29.53 |
| 175.28 | 21.33 | -0.09 | 9.91 | 522.78 | 45.11 | -3.28 | 30.70 |
| 178.34 | 21.93 | -0.05 | 10.10 | 747.78 | 49.55 | -4.66 | 35.56 |
| 181.80 | 22.64 | -0.02 | 10.49 | 780.74 | 50.20 | -4.82 | 36.33 |
| 185.24 | 23.30 | -0.04 | 11.27 | 810.04 | 50.78 | -5.00 | 36.91 |
| 189.06 | 24.06 | -0.12 | 11.46 | 847.58 | 51.52 | -5.23 | 37.50 |
| 192.68 | 24.76 | -0.14 | 12.24 | 892.50 | 52.42 | -5.52 | 38.47 |
| 196.28 | 25.49 | -0.18 | 12.63 | 924.80 | 53.07 | -5.73 | 39.25 |
| 199.78 | 26.19 | -0.29 | 13.21 | 998.56 | 54.52 | -6.30 | 41.00 |
| 203.84 | 26.98 | -0.34 | 13.60 | 1086.20 | 56.25 | -7.05 | 41.58 |
| 207.56 | 27.73 | -0.41 | 14.18 | 1285.04 | 60.20 | -9.08 | 46.82 |
| 211.50 | 28.48 | -0.48 | 14.77 | 1322.80 | 60.95 | -9.58 | 47.21 |
| 215.22 | 29.22 | -0.59 | 15.35 | 1350.00 | 61.49 | -9.94 | 48.38 |
| 219.16 | 30.01 | -0.69 | 16.13 | 1382.42 | 62.19 | -10.41 | 48.57 |
| 223.02 | 30.78 | -0.80 | 16.71 | 1411.12 | 62.70 | -10.89 | 49.74 |
| 226.88 | 31.55 | -0.87 | 17.68 | 1438.96 | 63.33 | -11.37 | 49.93 |
| 230.60 | 32.29 | -1.07 | 17.87 | 1479.26 | 64.07 | -11.98 | 50.52 |
| 234.48 | 33.05 | -1.16 | 18.65 |  |  |  |  |
| $\sigma_{c}=P_{\max}/S$ $E=\sigma_{h(50)}/\varepsilon_{h(50)}$ $\mu=-\varepsilon_{r(50)}\varepsilon_{h(50)}$ | | | | | | | |

**Table. Stress - strain data sheet of Sample C4 under uniaxial compression**

| Time（s） | Axial strain  $\varepsilon_{h}$（$\times10^{-4}$） | Radial Strain$\varepsilon_{r}$（$\times10^{-4}$） | Axial stress  $\sigma_{h}$（MPa） | Time（s） | Axial strain  $\varepsilon_{h}$（$\times10^{-4}$） | Radial Strain$\varepsilon_{r}$（$\times10^{-4}$） | Axial stress  $\sigma_{h}$（MPa） |
| --- | --- | --- | --- | --- | --- | --- | --- |
| 0.00 | 0.00 | 0.00 | 0.00 | 233.06 | 40.71 | -2.71 | 19.27 |
| 10.00 | 0.29 | -0.15 | 0.77 | 239.58 | 42.15 | -3.13 | 19.27 |
| 17.86 | 0.76 | -0.21 | 1.16 | 250.34 | 44.09 | -3.57 | 22.16 |
| 27.52 | 1.61 | -0.48 | 1.35 | 256.62 | 45.32 | -3.94 | 23.12 |
| 37.66 | 3.08 | -0.13 | 1.35 | 263.52 | 46.72 | -4.32 | 24.47 |
| 48.30 | 4.89 | -0.56 | 1.35 | 270.14 | 48.02 | -4.75 | 25.63 |
| 57.16 | 6.68 | -0.23 | 2.12 | 276.86 | 49.31 | -5.17 | 26.79 |
| 65.00 | 8.46 | -0.62 | 2.31 | 282.94 | 50.70 | -5.73 | 26.98 |
| 76.90 | 10.36 | -0.38 | 2.89 | 316.78 | 52.85 | -6.63 | 28.91 |
| 87.84 | 12.27 | -0.02 | 3.66 | 390.12 | 54.23 | -6.97 | 29.29 |
| 108.58 | 16.12 | -0.21 | 4.62 | 463.10 | 55.53 | -7.44 | 30.64 |
| 122.66 | 18.91 | -0.17 | 5.59 | 528.30 | 56.88 | -7.97 | 31.80 |
| 138.86 | 22.39 | -0.15 | 6.55 | 593.32 | 58.21 | -8.65 | 32.57 |
| 150.48 | 24.38 | -0.31 | 8.09 | 707.42 | 60.34 | -9.80 | 34.69 |
| 158.48 | 25.94 | -0.42 | 8.87 | 776.74 | 61.68 | -10.68 | 36.04 |
| 175.62 | 29.40 | -0.77 | 10.79 | 847.18 | 63.08 | -11.72 | 36.81 |
| 182.34 | 30.82 | -0.94 | 11.76 | 914.14 | 64.54 | -12.85 | 37.39 |
| 190.40 | 32.20 | -1.19 | 12.72 | 991.96 | 65.94 | -14.12 | 38.35 |
| 196.78 | 33.54 | -1.36 | 13.87 | 1062.60 | 67.41 | -15.49 | 39.89 |
| 206.52 | 35.64 | -1.73 | 14.65 | 1140.06 | 68.90 | -17.10 | 41.05 |
| 213.20 | 36.77 | -1.90 | 16.38 | 1219.72 | 70.49 | -18.89 | 41.43 |
| 219.70 | 38.15 | -2.17 | 16.77 | 1290.90 | 71.90 | -20.77 | 42.40 |
| 227.26 | 39.53 | -2.42 | 18.31 | 1410.40 | 74.37 | -24.59 | 43.55 |
| $\sigma_{c}=P_{\max}/S$ $E=\sigma_{h(50)}/\varepsilon_{h(50)}$ $\mu=-\varepsilon_{r(50)}\varepsilon_{h(50)}$ | | | | | | | |

**Table. Stress - strain data sheet of Sample C3 under uniaxial compression**

| Time（s） | Axial strain  $\varepsilon_{h}$（$\times10^{-4}$） | Radial Strain$\varepsilon_{r}$（$\times10^{-4}$） | Axial stress  $\sigma_{h}$（MPa） | Time（s） | Axial strain  $\varepsilon_{h}$（$\times10^{-4}$） | Radial Strain$\varepsilon_{r}$（$\times10^{-4}$） | Axial stress  $\sigma_{h}$（MPa） |
| --- | --- | --- | --- | --- | --- | --- | --- |
| 0.00 | 0.00 | 0.00 | 0.00 | 1483.22 | 90.04 | -91.60 | 30.88 |
| 8.84 | 0.13 | -0.09 | 1.17 | 1487.68 | 90.11 | -93.44 | 29.51 |
| 98.00 | 15.96 | -0.54 | 3.52 | 1488.76 | 90.17 | -94.14 | 28.14 |
| 119.58 | 20.07 | -0.29 | 4.10 | 1490.46 | 90.15 | -94.66 | 30.29 |
| 141.90 | 24.48 | -0.18 | 5.28 | 1491.60 | 90.20 | -95.36 | 27.95 |
| 155.36 | 27.12 | -0.34 | 6.25 | 1493.22 | 90.22 | -95.94 | 29.90 |
| 172.66 | 30.51 | -0.57 | 7.23 | 1494.34 | 90.32 | -96.72 | 27.75 |
| 209.76 | 37.81 | -4.12 | 10.55 | 1495.80 | 90.31 | -97.15 | 29.71 |
| 229.40 | 41.67 | -3.55 | 12.51 | 1497.18 | 90.28 | -98.09 | 27.75 |
| 239.68 | 43.63 | -1.80 | 13.09 | 1498.36 | 90.33 | -98.55 | 29.12 |
| 248.18 | 45.42 | -2.86 | 14.07 | 1501.18 | 90.42 | -100.25 | 27.95 |
| 258.50 | 47.36 | -2.51 | 15.05 | 1502.84 | 90.40 | -100.83 | 29.31 |
| 264.82 | 48.63 | -1.24 | 16.03 | 1504.54 | 90.48 | -102.10 | 26.97 |
| 271.18 | 49.88 | -0.53 | 17.20 | 1506.18 | 90.47 | -102.89 | 29.31 |
| 279.36 | 51.48 | -1.78 | 18.18 | 1514.30 | 90.65 | -116.63 | 27.17 |
| 291.20 | 53.82 | -0.38 | 19.74 | 1514.74 | 90.69 | -118.34 | 27.75 |
| 300.52 | 55.62 | -0.65 | 20.52 | 1514.76 | 90.69 | -118.45 | 27.75 |
| 311.48 | 57.80 | -1.13 | 22.28 | 1535.14 | 91.24 | -135.65 | 27.36 |
| 317.58 | 58.98 | -1.47 | 23.26 | 1535.24 | 91.20 | -135.65 | 25.99 |
| 329.06 | 61.26 | -2.06 | 25.02 | 1535.74 | 90.99 | -135.60 | 23.50 |
| 344.34 | 64.23 | -3.24 | 27.17 | 1535.98 | 91.03 | -135.70 | 25.02 |
| 355.28 | 66.42 | -4.00 | 28.14 | 1559.90 | 91.65 | -143.96 | 26.97 |
| 376.48 | 68.32 | -5.29 | 29.12 | 1559.94 | 91.64 | -144.02 | 26.38 |
| 518.54 | 71.12 | -6.45 | 30.10 | 1567.44 | 91.76 | -146.49 | 27.17 |
| 634.70 | 73.49 | -7.96 | 31.27 | 1567.70 | 91.78 | -146.56 | 26.19 |
| 738.10 | 75.56 | -9.93 | 32.44 | 1570.90 | 91.82 | -147.66 | 26.77 |
| 891.70 | 78.40 | -14.10 | 33.03 | 1570.92 | 91.80 | -147.68 | 26.77 |
| 918.38 | 78.95 | -15.27 | 33.81 | 1573.60 | 91.91 | -148.62 | 27.17 |
| 958.98 | 79.78 | -17.04 | 34.00 | 1573.82 | 91.91 | -148.82 | 25.60 |
| 1480.24 | 89.96 | -90.51 | 31.27 |  |  |  |  |
| $\sigma_{c}=P_{\max}/S$ $E=\sigma_{h(50)}/\varepsilon_{h(50)}$ $\mu=-\varepsilon_{r(50)}\varepsilon_{h(50)}$ | | | | | | | |
